# Nano-enabled agrochemicals: mitigating heavy metal toxicity and enhancing crop adaptability for sustainable crop production

**DOI:** 10.1186/s12951-024-02371-1

**Published:** 2024-03-05

**Authors:** Abazar Ghorbani, Abolghassem Emamverdian, Necla Pehlivan, Meisam Zargar, Seyed Mehdi Razavi, Moxian Chen

**Affiliations:** 1 National Key Laboratory of Green Pesticide, Key Laboratory of Green Pesticide and Agricultural Bioengineering, Ministry of Education, Center for R&D of Fine Chemicals of Guizhou University, Guiyang, 550025 China; 2 Department of Biology, Faculty of Sciences, University of Mohaghegh Ardabili, Ardabil, Islamic Republic of Iran; 3 Co-Innovation Center for Sustainable Forestry in Southern China, Nanjing Forestry University, Nanjing, 210037 China; 4 Biology Department, Faculty of Arts and Sciences, Recep Tayyip Erdogan University, Rize, 53100 Türkiye; 5 Department of Agrobiotechnology, Institute of Agriculture, RUDN University, Moscow, 117198 Russia

**Keywords:** Nanoparticles, Agrochemical, Heavy metal stress, Sustainable agriculture

## Abstract

The primary factors that restrict agricultural productivity and jeopardize human and food safety are heavy metals (HMs), including arsenic, cadmium, lead, and aluminum, which adversely impact crop yields and quality. Plants, in their adaptability, proactively engage in a multitude of intricate processes to counteract the impacts of HM toxicity. These processes orchestrate profound transformations at biomolecular levels, showing the plant’s ability to adapt and thrive in adversity. In the past few decades, HM stress tolerance in crops has been successfully addressed through a combination of traditional breeding techniques, cutting-edge genetic engineering methods, and the strategic implementation of marker-dependent breeding approaches. Given the remarkable progress achieved in this domain, it has become imperative to adopt integrated methods that mitigate potential risks and impacts arising from environmental contamination on yields, which is crucial as we endeavor to forge ahead with the establishment of enduring agricultural systems. In this manner, nanotechnology has emerged as a viable field in agricultural sciences. The potential applications are extensive, encompassing the regulation of environmental stressors like toxic metals, improving the efficiency of nutrient consumption and alleviating climate change effects. Integrating nanotechnology and nanomaterials in agrochemicals has successfully mitigated the drawbacks associated with traditional agrochemicals, including challenges like organic solvent pollution, susceptibility to photolysis, and restricted bioavailability. Numerous studies clearly show the immense potential of nanomaterials and nanofertilizers in tackling the acute crisis of HM toxicity in crop production. This review seeks to delve into using NPs as agrochemicals to effectively mitigate HM toxicity and enhance crop resilience, thereby fostering an environmentally friendly and economically viable approach toward sustainable agricultural advancement in the foreseeable future.

## Introduction

**Fig. 1:**
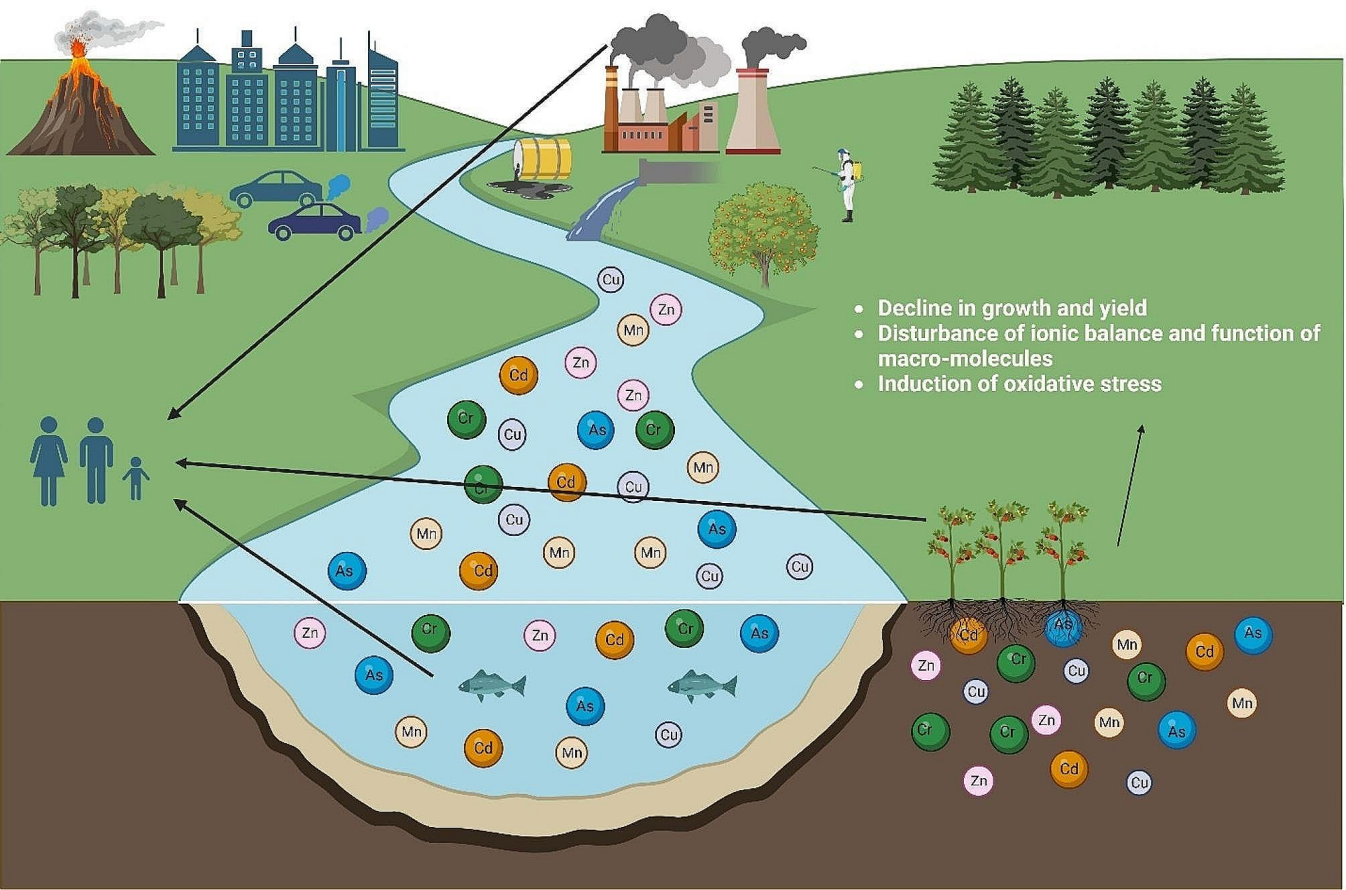
The origins of heavy metal (HM) toxicity occurrence, which negatively affects the development and productivity of crops and presents a significant risk to human health due to HM entrance into the food chain (Inspired by Fakhar et al. [242])

Despite being the main driver of society and economic development, industrialization has also resulted in the release and buildup of numerous environmental pollutants in ecosystems, raising the risks for people, plants, and other living things [1]. Due to their toxicity and longevity in ecosystems, heavy metals (HMs) are a class of pollutants that warrants substantial attention among other pollutants. HMs have metallic properties with an atomic density above 3 g/cm^3^ and an atomic number bigger than 20, encompassing a selection of semi-metals, transition metals, actinides, and lanthanides [2]. Although HMs are abundant in the earth’s crust, they are hardly accessible to humans and plants in their geochemical form. However, anthropogenic activities, e.g., using fertilizers, smelting, sewage sludge, industrial waste disposal, and disposal, have increased bioavailability to organisms (Fig. 1). The majority of the known HMs exhibit severe toxicity towards both flora and fauna, with significant examples e.g., cadmium (Cd), lead (Pb), arsenic (As), and nickel (Ni) (1). Also, HMs easily navigate the food chain due to their persistence in the environment and their inherent resistance to biodegradation. These powerful characteristics pose a severe risk to human health, justifying our utmost concern. Therefore, besides their roles as trace nutrients in biological systems, where they are needed in minimal amounts, most HMs are harmful to life, even in very low quantities [3].

In the last decade, the security of crop production has been seriously threatened by the enhancement in the contamination of arable land with toxic HMs [3]. Research has demonstrated that the detrimental effects of high HM concentrations on crops are due to the impairment of bio-macromolecules, disruption of ion balance, excessive reactive oxygen species (ROS) generation and toxic radicals/induction of oxidative stress, and damage to photosynthetic apparatus. Various approaches, e.g., marker-mediated breeding, transgenic crop engineering, and conventional breeding, have been created to lower the effects of HMs on crops [3, 4]. On the other hand, in recent years, nano-based techniques have emerged as highly promising approaches to regulating abiotic stresses induced by the environment, enhancing agricultural productivity, addressing nutrient deficiencies, and revolutionizing biological systems [5]. In addition, there has been considerable interest in utilizing nanoparticles (NPs) as a targeted delivery of micronutrients in an environmentally sustainable and economical manner, as opposed to chemical fertilizers [6]. Recently, many techniques have been relied on to synthesize metallic NPs, such as chemical (co-precipitation, micro-emulsion, sol-gel, hydrothermal), physical (ion sputtering scattering, gamma radiations, electro-explosion, ion sputtering, pulse laser ablation, mechanical/ball milling), and biological (green synthesis with microorganisms and plant extracts) approaches [7]. According to the latest research, the external application of NPs can enhance the adaptation of plants to HMs by adjusting morpho-physiological and molecular mechanisms [8–11]. Hence, in sustainable agriculture, using NPs to stimulate plant adaptation to HM toxicity can be regarded as a reliable, ecologically safe, and long-term effective method, in contrast to conventional methodologies.

Here, we have reviewed the latest avenues in applying nanomaterials as protective agrochemicals in reducing HM toxicity and their detailed function in modulating plant defense mechanisms, along with practical recommendations for future studies to develop better strategies in sustainable agriculture.

## Potential role of nanomaterials in heavy metal toxicity tolerance

**Fig. 2:**
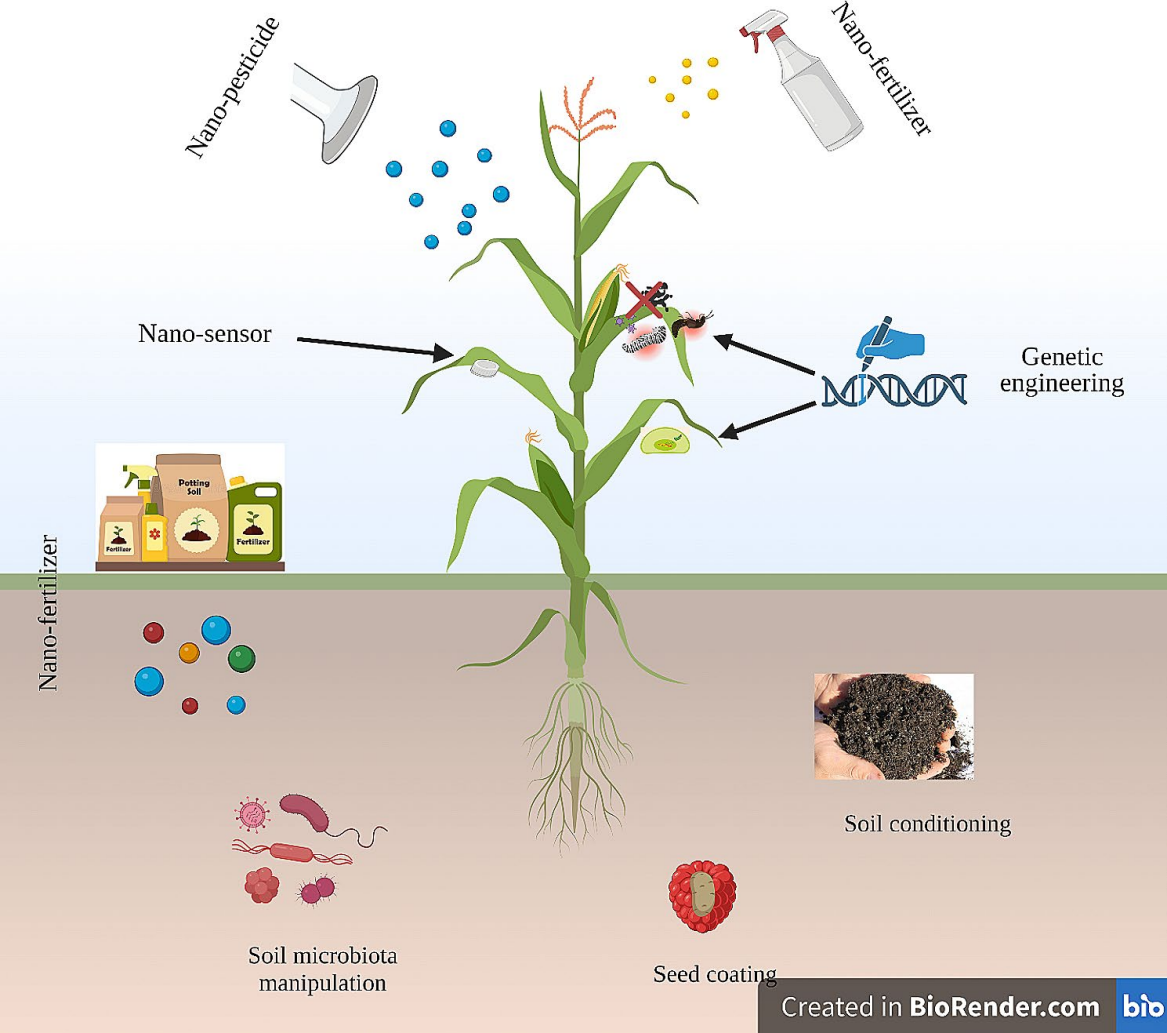
Potential applications of nanoparticles in agriculture

**Fig. 3:**
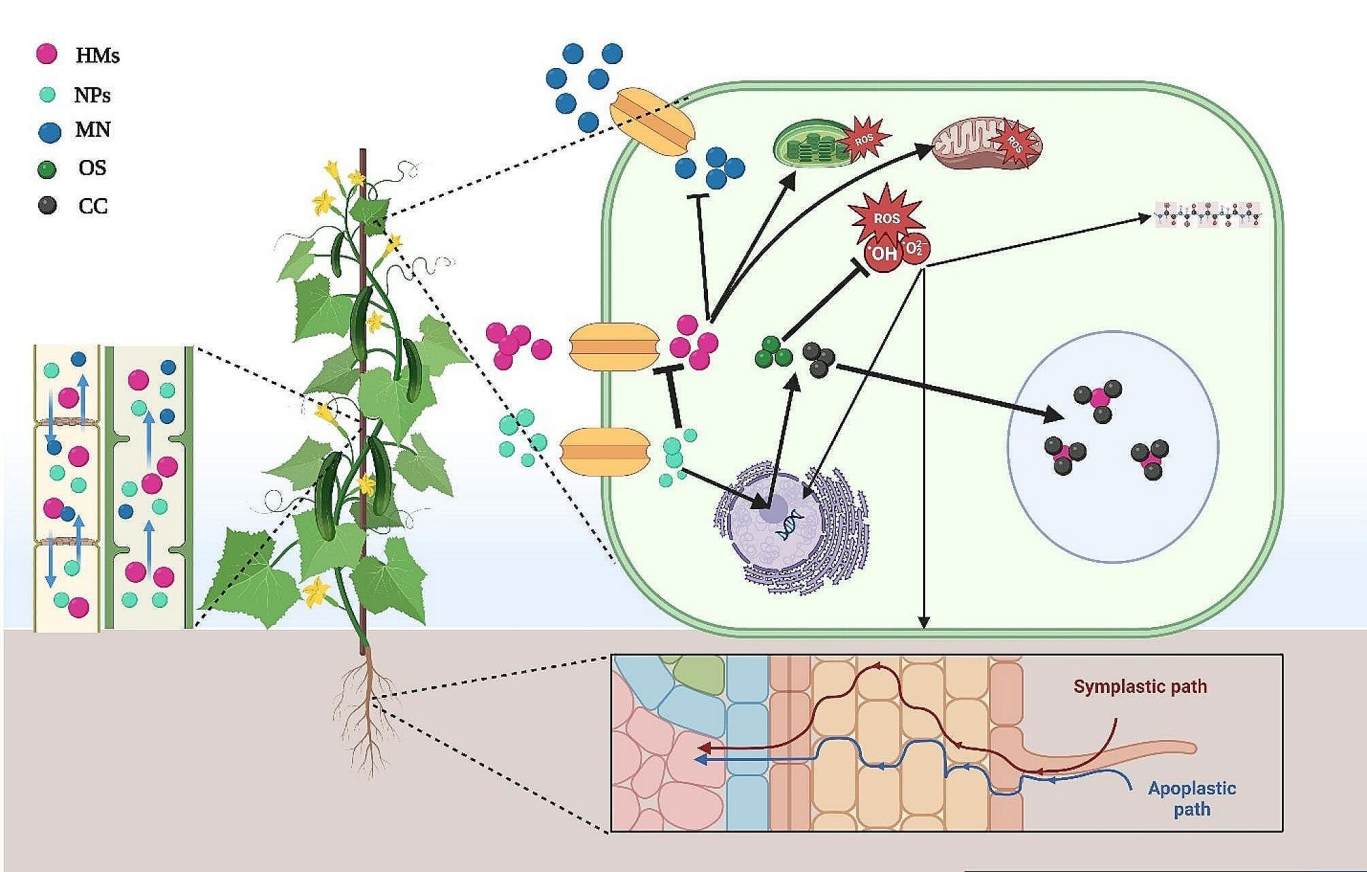
Schematic representation of the interaction of nanoparticles (NPs) with heavy metals (HMs) in plants. After being absorbed and passing through the apoplastic and symplastic pathways, HMs are transported to different organs by the xylem and phloem tissues. HMs prevent the entry of mineral nutrients (MN) into cells, disrupt ion homeostasis, cause toxic radical accumulation, and induce oxidative stress by disrupting the function of the cell’s vital processes, e.g., photosynthesis and respiration, which damage bio-macromolecules such as proteins, lipids, and DNA. By reducing the absorption of HMs, NPs improve the cellular ionic balance and diminish HMs-induced oxidative stress by triggering osmolyte (OS) (proline, glycine betaine, and soluble sugars) accumulation. NPs also increase chelator compounds (CC) as phytochelatins and glutathione, transferring the CC-HMs complex to the vacuole and counteracting HM toxicity

Nanotechnology is widely acknowledged for its beneficial uses in enhancing crop quality and yield. These applications include its potential in alleviating abiotic stressors and mitigating the impacts of climate change [4]. The key nano-based technologies utilized to enhance crop growth and yield include the implementation of nanosensors for monitoring the quick nutritional condition of plants, administering nano fertilizers via foliar spray, soil irrigation, seed coating, or and genetic engineering to enhance the photosynthetic efficiency and disease resistance (Fig. 2). The utilization of NPs offers several benefits in comparison to traditional fertilizers. These advantages stem from their ability to efficiently provide vital minerals as nanofertilizers to plants and soil, superior efficacy in eliminating contaminants, and high surface-area-to-volume ratio [12, 13]. In response to abiotic stress, particularly HM toxicity, several studies have shown that nanomaterials enhance plant adaptability and boost agricultural yields [14, 15] of crops by supplying necessary nutrients/reducing nutrient losses due to their large surface area and nutrient-holding capacity [16], by regulating phytohormone levels, enhancing photosynthetic performance, boosting antioxidant defense, and reducing the uptake of HMs (Fig. 3). The NP nutrient-holding capacity is related to their physical (such as carbon-based NPs containing tiny pores capable of absorbing and retaining soil micronutrients) and chemical properties (such as multi-nutrient NPs including NPK-NPs) [243]. Adrees et al. [17] noted that the adaptation of wheat plants subjected to Cd treatment were enhanced through the Fe-NPs. The biomass of rice seedlings treated to As treatment benefited through the external application of Si- and TiO_2_-NPs, which modulated the expression of As transporters and strengthened the antioxidant system [18]. The use of NPs in various crops subjected to abiotic stress has been associated with several advantages, including better growth and adaptation in wheat [19], corn [20], barley [21], rice [8, 22], and tomato [23]. Hence, taking into account the capacity of NPs to hinder the harmful impact of HMs, NPs can address a multitude of adverse effects resulting from toxicity in agriculture.

## Impacts of NPs on plants under HM toxicity

### Impacts of NPs on plants under as toxicity

#### Uptake, transport, and metabolism of as

**Fig. 4:**
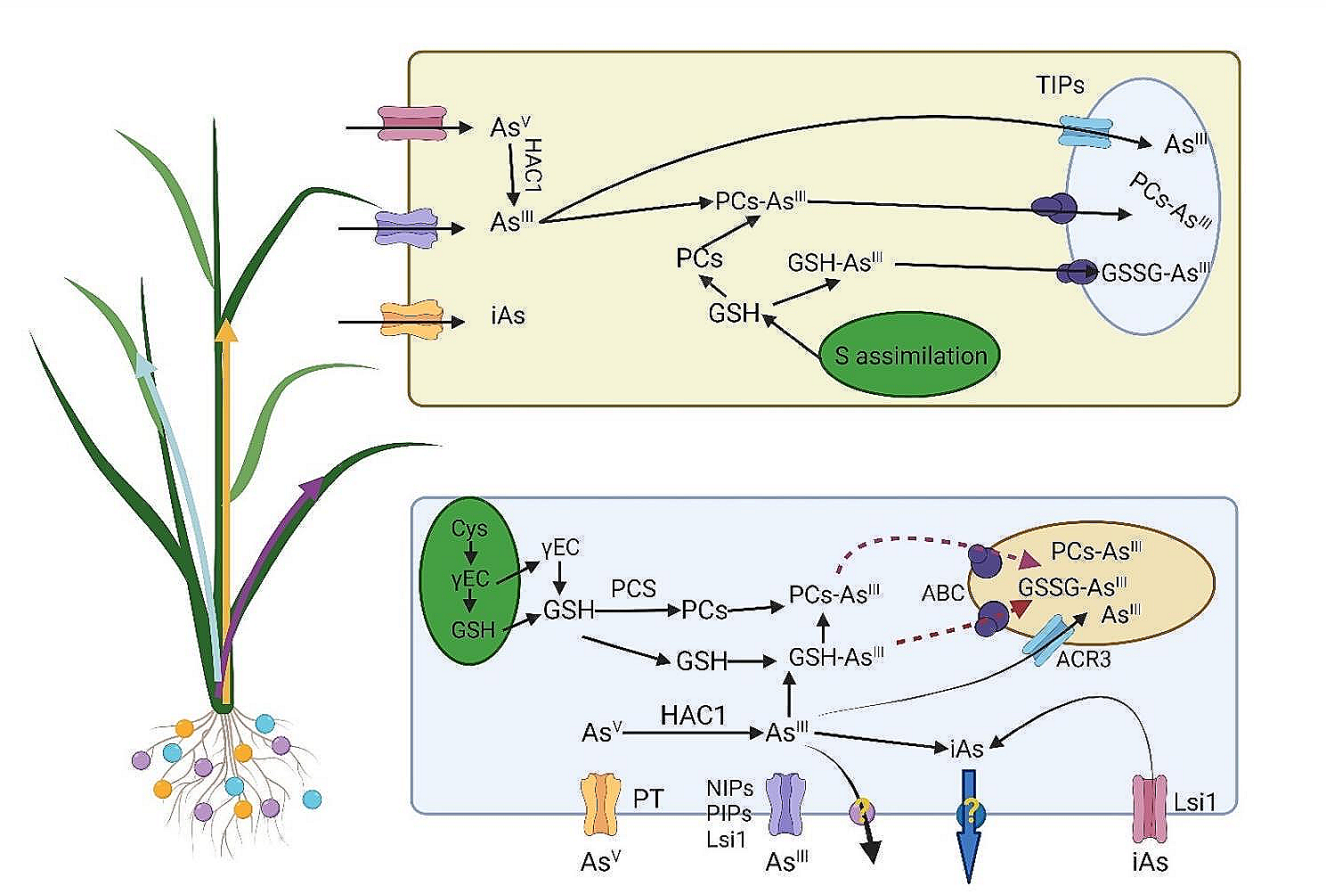
Schematic illustration of plants’ arsenic (As) uptake, transport, and metabolism

The predominant forms of As found in soil systems, which plants may readily absorb, include organic molecules (dimethylarsinic acid (DMA) and mono-methylarsonic acid (MMA)) as well as inorganic compounds like As^III^ and arsenate (As^V^). Under circumstances lacking oxygen, As^III^ prevails as the primary type of As in the soil. It penetrates the roots via membrane channels, such as aquaporins. Studies in Arabidopsis have demonstrated that the NIP subfamily, which consists of NIP3;1, NIP1;2, NIP1;1, NIP6;1, NIP5;1, and NIP7;1, serve an integral part in facilitating the transport of As^III^ to the roots [24, 246]. NIP2;1 (Lsi1) and NIP2;2 (Lsi2) play a role in the absorption of As^III^ and its transportation to the xylem tissue in rice plants [25]. Indeed, a portion of the As^III^ that reaches roots subsequently transport back to the rhizosphere by specific transporters, including NIP3;1, NIP3;2, NIP2;1, and NIP1;1 [26]. Other plant subfamilies of aquaporins that contribute to the transport and accumulation of As^III^ include small basic intrinsic proteins, plasma membrane intrinsic proteins (PIP), tonoplast intrinsic proteins (TIPs), and uncategorized intrinsic proteins (XIPs) [27] (See Fig. 4).

As^V^ enters the roots via phosphate transporters and competes with inorganic phosphate as it does in aerobic conditions, where it is its dominant form. Several phosphate transporters were identified in Arabidopsis plants that participate in As^V^ uptake into roots, including PHT 1;1, 1;4, 1;5, and 1;8 [28]. In rice plants, silencing the expression of phosphate transporters (PT1, PT4, and PT8) decreased AsV absorption, and their overexpression increased AsV absorption, indicating these transporters’ participation in AsV absorption and transport [28, 29]. Organic forms of As (DMA and MMA) accumulate in plant cells at a lower rate than inorganic forms. Carey et al. [30] showed that compared to As^III^, MMA is transported to grains more efficiently (more than 10 times) through the phloem and xylem. It has been shown that in rice grains, organic forms of As accumulate more than inorganic ones [31]. While the process of absorption and transport of organic forms of As in plants is not well known, recent studies have shown that *OsLsi1* may participate in the transport of DMA/MMA [32] (See Table 1).

**Table 1:** The list of different nanoparticle (NP)s used in different plant species under arsenic (As) toxicity

NP	NP concentration	Plant species	Details	Ref
ZnO	10 ~ 200 mg/L	*Triticum aestivum, Oryza sativa, Luffa acutangula, Zea mays, Glycine max*	Improved growth, germination rate, proline, photosynthetic pigments, PCs content, net photosynthetic rate, stomatal conductance, ionic homeostasis, Adjusted the internal level of phytohormones, Upregulated the activity of antioxidant enzymes, Declined As uptake, MDA and ROS levels	[14, 46, 48, 50, 214–216]
FeO	5 ~ 100 mg/L	*Oryza sativa, Cucurbita moschata, Cucumis melo*	Enhanced growth, germination, GSH, PCs, gas exchange attributes, antioxidant enzymes and glyoxalase system, Decreased the expression of *Lsi1* and *Lsi2*, Upregulated the expression of genes involved in Fe absorption and transport (*IRT1*, *IRT2*, *YSL2*, *YSL13*, *FRDL1*, *DMAS1*, *NAS2*, and *NAS3*), Enhanced the biosynthesis of spermidine and putrescine, Regulated expression of *respiratory burst oxidase homologue D* (*RBOHD*), *chlorophyll synthase* (*CHLG*) and *protochlorophyllide oxidoreductase* (*POR*), Reduced H_2_O_2_, MDA, and EL levels, and As uptake	[8, 217–219]
Fe_2_O_3_	100 ~ 1000 mg/L	*Vigna radiate, Glycine max*	Improved the biomass and photosynthesis-related traits, Reduced H_2_O_2_ and MDA, Induced CAT, SOD, and GPX activity, Increased proline level, Reduced As uptake	[52, 53]
Fe_3_O_4_	500 mg/L	*Brassica juncea*	Improved parameters of germination, Reduced the activity of CAT, SOD, and APX, Modulated the expression of genes involved in S assimilation (*APS*, *APR*, *LAST*, *O-ASL*)	[220]
TiO_2_	10 ~ 1000 mg/L	*Oryza sativa, Vigna radiata*	Improved growth indices, antioxidant enzymes, glyoxalase cycle, the content of GSH and PCs, Reduced As absorption, H_2_O_2_, MG, MDA, and EL, Modulated the expression of Si/As transporters (*Lsi1*, *Lsi2*, and *Lsi6*), Upregulated the expression of *CAT*, *SOD, GSH1*, *PCS*, and *ABC1*, Declined iron plaque on the root surface	[18, 58, 59]
CaO	25 mg/L	*Hordeum vulgare*	Improved growth and biomass, Reduced As accumulation and ROS, Upregulated the activity of CAT, POD, and SOD, Downregulated the expression of phosphate transporter genes	[10]
MgO	50 ~ 200 mg/L	*Oryza sativa, Glycine max*	Increased biomass, photosynthesis, nutrient uptake, and activity of CAT and SOD, Reduced As, MDA, and H_2_O_2_ accumulation	[16, 221]
Mo	100 mg/L	*Triticum aestivum*	Augmented biomass and height, Improved ion balance, Diminished As absorption	[222]
SiO_2_, Si-doped biochar	50 ~ 2000 mg/L	*Zea mays, Solanum lycopersicum, Oryza sativa, Chenopodium quinoa*	Increased the growth, photosynthetic pigments, antioxidant enzyme activity, components of the AsA-GSH cycle, glyoxalase cycle, PCs content, Decreased As uptake and translocation, H_2_O_2_, MG, MDA, and EL, Upregulated the expression of *GSH1*, *PCS*, and *ABC1* genes	[54, 55, 223, 224]
MWCNTs	100 ~ 1000 mg kg		Increased the shoot length, biomass, antioxidant enzymatic activities, micronutrient content, and the accumulation of As, Reduced bioavailable As in rhizosphere and bio-concentration factor of As	[62]

The first step in the metabolism of As in the direction of detoxification includes the enzymatic and non-enzymatic process of converting As^V^ to As^III^. In the enzymatic pathway, the arsenate reductase (AR) enzyme converts As^V^ to As^III^ [33]. Recent research in the roots of *A. thaliana* identified a novel arsenate tolerance variant QTL1 that promotes the conversion of As^V^ to As^III^ [34]. Bleeker et al. [35] claimed that the CDC25-proteins and As compounds resistance 2 (ACR2) were implicated in the reduction of As^V^. HAC1, an arsenite reductase, has been demonstrated to inhibit the transfer of arsenite to the shoot of Arabidopsis by converting As^V^ to As^III^ and thereby decreasing the As^V^ accumulation in the roots [36]. According to Chao et al. [34], HAC1 dysfunction increases As^III^ translocation to shoots while decreasing As^III^ efflux in roots. Arsenite reductase HAC1 has been shown to inhibit the transfer of AsV to the Arabidopsis shoot by changing AsV into As^III^, which reduces the amount of As^V^ that builds up in the roots. The overexpression of HACs genes was associated with the decrease of As accumulation in the root resulting from the increase of As^III^ efflux. Sulfhydryl-based complexes help assimilate the majority of As^III^, which is still present in root cells [37]. Increased accumulation of glutathione (GSH), the main precursor of phytochelatins (PCs), is one of the primary detoxification responses of plants against accumulated AsIII. By creating As-complexes and keeping them in vacuoles via the ABCC1 and ABCC2 transporters, raising the amount of GSH and PCs enhances the cell’s capacity to detoxify cytosolic As. Two known PC synthase (PCS) enzymes, PCS1 and PCS2, biosynthesize PCs. Glutathione S-transferases (GST) have also been demonstrated to facilitate the formation of complexes between GSH and As^III^ [38]. Furthermore, inorganic As methylation occurs in plant tissues alongside As^V^ to As^III^ reaction. The organic species (DMA and MMA) were generated in tissues following exposure to As^V^ and As^III^ treatments [38]. However, the literature lacks comprehensive details regarding As methylation pathways or the enzymes participating in this pathway. Only in eukaryotic algae has the enzyme As-methyltransferase (ASMT) been identified, which uses GSH to methylate AsIII and produce DMA and MMA [39].

#### The role of engineered nanoparticles in arsenic detoxification

As is a widely dispersed, extremely toxic metalloid that is dangerous to health even at low concentrations. A Class I human carcinogen, according to Rehman et al. [40]. As primarily enters the ecosystem via geogenic or natural processes, including mining operations and rock weathering, as well as anthropogenic activities, including the combustion of fossil fuels. As is predominantly found in arsenate (+ V), arsenite (+ III), arsine (-III), and arsenic (0), oxidation states in the environment [41]. By their capacity to inhibit biological processes via interaction with thiol groups in proteins, inorganic forms of As are more toxic than their organic counterparts [42]. On the contrary, research has demonstrated that As^III^ is more toxic to plants than As^V^, primarily due to its elevated solubility in water [43]. Overall, As causes major damage to the morpho-physiological aspects of several plant species [44]. At the morpho-physiological and metabolic stages, As leads to a decline in germination, leaf wilting, and biomass and disrupts the water balance. It also hinders chlorophyll metabolism, adversely affects critical metabolic functions such as reproduction, respiration, and photosynthesis, and obstructs the uptake of vital nutrients, ultimately resulting in a decline in growth. At the biochemical levels, the overproduction of ROS induced by As is highly detrimental to plants. This is because ROS can severely disrupt plant metabolisms and lead to oxidative damage in important bio-macromolecules like carbohydrates, proteins, nucleic acids, and lipids [44, 247, 248]. Nanotechnology has emerged as a significant asset for advancing plant defenses against environmental challenges in the modern era. As evidenced by many studies, exogenous administration of various NPs mitigates the toxicity of As^III^ or As^V^ to different plant species.

##### Zinc oxides (ZnO) NPs

ZnO-NPs have been used for decades to supply vital nutrients to crops in areas lacking in Zn. Furthermore, by lessening the accumulation and toxicity of HMs, Zn can enhance physiological processes and plant development. The overall As in rice plants was significantly reduced when ZnO-NPs were used with As^III^ or As^V^ [45]. Furthermore, ZnO-NPs have demonstrated greater efficacy in declining the absorption of As^III^ in rice plants over As^V^. Hence, the reducing effects of ZnO-NPs on inorganic As forms were more significant in the root system than in the plants’ upper portions. This suggested that rather than altering the movement of As from roots to shoots, ZnO-NPs had a larger function on lowering As accumulation. The possible cause for the decline in As accumulation triggered by ZnO-NPs may be attributed to ROS production, as documented in studies on *A. thaliana* [12]. The production of ROS may result in the oxidation of chemicals produced by the roots, disrupting the flow of electrons. This disruption is crucial for forming inorganic forms of As in plants. Remarkably, combining ZnO-NPs with As^III^ resulted in greater total As in the shoots over As^V^ combination treatments. This indicates that the two inorganic forms of As followed distinct pathways for accumulation and transportation within rice plants. Moreover, subsequent findings have shown that ZnO-NPs lowered As levels in *Luffa acutangula*. This exposure resulted in advantages in photosynthetic qualities, nutrient absorption, abscisic and auxin hormones, antioxidant enzyme activity, and total soluble sugars [46].

As uptake and accumulation have been effectively reduced by utilizing ZnO-NPs, according to studies [45, 47, 48]. The decline in the overall concentration of As in plant shoots can be ascribed to the mechanism by which Zn inhibits the formation of organic As compounds and/or AsV in the roots. Roots may have lower levels of As^V^ because they are converted to As^III^, which is a possible explanation for the lower levels of As^V^ observed in roots [49]. In addition, some reports have shown that 10–100 mg/L ZnO-NPs significantly increased germination rates, early growth stages, Zn supply, chlorophyll content, and antioxidant defense enzymes` activity. The benefits above are attained through the reduction of As, MDA, and ROS accumulation [50]. As a result of the enhanced adsorption capacity of ZnO-NPs, As levels in the nutrient solution were considerably reduced, leading to a decrease in As accumulation in the roots. Therefore, ZnO-NPs may enhance Zn content and reduce As uptake, enhancing plant growth characteristics and As detoxification. This results in a reduction of As transport capacity within the cells. Furthermore, in the presence of ZnO-NPs, higher levels of PCs in roots result in the buildup of complexes with As^III^, which hinders the movement and mobility of As^III^ [14].

The drop in As levels in plant shoots can also be linked to the release of Zn by ZnO-NPs, reducing As concentrations. Moreover, the inverse relationship between Zn and As concentrations suggests that ZnO-NPs can notably reduce As levels in both plant roots and shoots [14]. ZnO-NPs not only raise the levels of Zn in agricultural products but also improve their nutritional quality. Thus, ZnO-NPs may be effectively employed as environmentally friendly agrochemicals to enhance plant growth. Therefore, it is highly suggested that the interactions between ZnO-NPs and inorganic As forms be thoroughly investigated during the plant’s entire life cycle under different soil matrixes. Also, additional data sets are necessary to identify possible benefits of treating seeds and exposing roots to ZnO-NPs in reducing As toxicity and unique mechanisms that regulate the detoxification. However, the ideal dose of externally treated ZnO-NPs to alleviate the harmful effects of As in various plant species has not yet been determined (See Table 2).

**Table 2:** The list of different nanoparticle (NP)s used in different plant species under Cd toxicity

NP	NP concentration	Plant species	Details	Ref
ZnO	1 ~ 300 mg/L	*Oryza sativa, Triticum aestivum, Zea mays, Leucaena leucocephala, Lactuca sativa*	Improved the biomass, yield, photosynthesis, ion homeostasis, RWC, chloroplast structure, Zn concentration, and antioxidant enzyme activity; modulated protein interactors (Metallo endo proteinase 1- and 5-MMP, Alpha-amylase, and Zn-dependent exo peptidase superfamily), diminished Cd uptake, MDA, and oxidative injuries	[9, 19, 82, 84, 85, 121, 122, 225, 226]
FeO	10 ~ 500 mg/L	*Triticum aestivum, Phaseolus vulgaris, Oryza sativa*	Increased growth, biomass, yield, net photosynthetic rate, gas exchange attributes, biosynthesis of polyamines, ionic homeostasis, and antioxidant activity, Reduced MDA, H_2_O_2_, ROS, EL, Cd uptake and translocation, and the expression of Cd transporter, *HMA2*, *HMA3*, and *LCT1*	[22, 117, 123, 227]
Fe_2_O_3_	100 mg/L	*Phaseolus lunatus*	Improved growth, Chl, photosynthetic efficiency, and RWC, Diminished MDA, MG, H_2_O_2_, and El	[124]
Fe_3_O_4_	10 ~ 100 mg/L	*Solanum lycopersicum*	Reduced Cd and ROS, Increased growth, nutrient intake, proline, free amino acids	[119]
TiO_2_	10 ~ 100 mg/kg	*Vigna unguiculata, Zea mays, Coriandrum sativum*	Increased growth, germination, gas exchange, RWC, photosynthetic pigments, levels of minerals and antioxidants, the activity of antioxidant enzymes, Reduced Cd uptake, MDA, H_2_O_2,_ and EL	[15, 118, 122]
SiO_2_	25 ~ 1200 mg/L	*Oryza sativa, Phaseolus vulgaris, Triticum aestivum, Satureja hortensis*	Enhanced growth, net photosynthetic rate, gas exchange attributes, biosynthesis of polyamines, concentrations of K, Mg, Fe, and Si, total phenolic and flavonoid content, and EO yield, Sequestered Cd in the cell wall, Diminished oxidative stress, Cd uptake, MDA, and EL	[88, 89, 117, 125, 228–230, 240]
CaO	25 mM	*Hordeum vulgare*	Increased the biomass, activities of APX, CAT, SOD, and GR, and the content of AsA and GSH; upregulated the expression of *Zn-SOD*, *CAT*, *APX*, *GR1* genes	[11]
Se	5 ~ 60 mg/L	*Coriandrum sativum, Brassica napus, Capsicum annuum*	Improved biomass, Chl, proline, RWC, phenolic and flavonoid contents, nutrients content, activity of CAT, APX and POX, and essential oil yield, Decreased MDA and Cd accumulation, Inhibiting the expression of NADPH oxidases (*RBOHC*, *RBOHD1*, and *RBOHF1*) and glycolate oxidase (*GLO*), oxidative stress, MDA, H_2_O_2_, and Cd accumulation, Improved intracellular Ca homeostasis, disulfide bond formation, and the waxy outer layer of the leaf surface	[86, 126, 127, 241]
Hydrogel	25 ~ 100 mg/kg	*Oryza sativa*	Increased biomass, antioxidant enzyme activity, photosynthesis, and nutrient acquisition, Declined ROS, Cd translocation, and the expression of Cd transporter, *HMA2*, *HMA3*, and *LCT1*	[22]
Ag	40 mg/L	*Daucus carota*	Declined ROS, MDA, and Cd uptake, Improved growth, Chl, and activity of POX, PPO, and PAL	[128]
Chitosan–Se	5–10 mg/L	*Dracocephalum moldavica*	Enhanced agronomic traits, photosynthetic pigments, chlorophyll fluorescence parameters, proline, phenols, antioxidant enzymes activities, Decreased MDA and H_2_O_2_	[231]
MWCNTs	100 ~ 1000 mg kg		Increased shoot length, biomass, antioxidant enzymatic activities, and micronutrient content, the accumulation of Cd, Reduced bioavailable Cd in the rhizosphere	[62]
CuO	5 ~ 100 mg/kg	*Triticum aestivum, Oryza sativa, Hordeum vulgare*	Increased growth, biomass, contents of N, P, K, and Ca, Enhanced activity and expression of SOD, POD, and CAT, Downregulated Cd-transporter genes (*Nramp5* and *HMA2*), Declined Cd uptake	[90, 184]

##### Iron oxide (fe) NPs

Fe-NPs have been utilized to purify water and As contaminated soil. Huang et al. [51] assessed the impact of these NPs on As accumulation. Both low and high quantities of Fe-NPs substantially impacted reducing As accumulation and its transportation to the above-ground sections of rice. The interplay between Fe-NPs at 100 to 400 mg/L and As at varying levels of 0.5 to 2 µM in *Vigna radiata* was reported by [52], and Fe-NPs produced a drop in the total As level in tissues. Additionally, Fe-NPs alleviated oxidative stress, decreased malondialdehyde, raised proline, boosted antioxidant capacity, and improved growth and seed germination rates. Furthermore, Fe-NPs contributed to preserving cellular viability during periods of As stress, confirming a decrease in the adverse effects of As. The NPs discharged a regulated quantity of Fe ions mostly to the roots, resulting in no adverse effects on plants. These hydroponic studies suggest that Fe-NPs may adsorb As on their surfaces, limiting its bioavailability for root uptake and consequently reducing As uptake and accumulation in plants. Therefore, using Fe-NPs to mitigate As toxicity in field conditions is recommended.

On the contrary, it was observed that Fe-NPs raised the absorption and transportation of Fe throughout the leaves of As-treated rice, which triggered the generation of photosynthetic pigments and overall growth. Bidi et al. [8] demonstrated that this beneficial impact on Fe uptake and transport was achieved via upregulating associated genes, including *DMAS1, IRT1, IRT2, FRDL1, YSL2*, and *NAS3*. The beneficial impacts of Fe-NPs were also detected in *Glycine max* when subjected to As stress; Fe-NPs augmented the photosynthetic pigments and growth characteristics. They also decreased H_2_O_2_ levels and MDA, contributing to antioxidant enzyme activity regulation. These helped restrict As entry into plant tissues, decreasing As toxicity [53]. Bidi et al. [8] reported that Fe-NPs trigger an increase in the concentration of chelators, such as PCs, GSH, and proline. The gradual accumulation of As in plant cells served as a defense mechanism by elevating As storage in vacuoles and binding As to cell walls, preventing harmful effects on cells. Fe-NPs also reduced cysteine content, a precursor to GSH, in leaves. This drop may be attributed to the stimulation of GSH synthesis. In addition, there was a simultaneous rise in GSH in leaves. These results validate the capacity of Fe-NPs to improve the process of storing As in vacuoles by increasing PC levels. Nevertheless, it is still uncertain if Fe-NPs directly impact the accumulation of PCs or if other elements or molecules also play a role in this process.

##### Silica (Si) NPs

Because conventional Si fertilizers have low bioavailability, they are seldom directly applied to plants. In contrast, Si-NPs can be directly applied to leaf surfaces because of their high bioavailability. When Si is deposited on cell walls, it reduces the phytotoxic effects caused by HMs [54]. Prior research has elucidated the mechanisms through which Si-NPs mitigate the toxicity of As. Experimental results from hydroponics revealed that Si-NPs decreased As levels in tomato seedlings. Furthermore, Si-NPs improved the antioxidant defense mechanism by inhibiting the ROS overproduction regulated by As, thereby mitigating lipid peroxidation in roots subjected to As stress [55]. It is evident that both Si and As are absorbed into plant tissues via analogous transporters; this shared mechanism is the most probable explanation for how Si-NPs decrease the availability of As. Si-NPs increase Si accumulation in cell walls, resulting in As retention within root tissue cells, as Cui et al. [56] observed. The movement of As to rice shoots and grains was impeded. Additionally, Si-NPs reduced the expression levels of *NIP1;1* and *NIP3;3* while increasing the transcript levels of *Lis1* and *Lis2* in roots. These results validated the role of Si-NPs in controlling the expression of genes associated with As and preventing its accumulation and movement in shoots and grains. Further research conducted in rice plants at the cellular level revealed that Si-NPs decreased As accumulation and associated toxicities by enhancing pectin content, cation exchange capacity, and cell wall thickness. Consequently, oxidative stress was diminished, viable cells increased, and cellular integrity was preserved [56]. On the other hand, Si-NPs elevated the expression of *GSH1* and *PCS*, which are accountable for the retention of metals within the vacuole. Concurrently, Si-NPs inhibited the Lsi1, Lsi2, and Lsi6 transporters, diminishing As uptake by roots and transferring assimilated As to leaves. This protective mechanism kept the leaves and photosynthetic process safe against As toxicity [18]. The quality of agricultural products can also be negatively affected by As. González-Moscoso et al. [244] demonstrated that Si-NPs, through altering antioxidant molecule compositions, improved the antioxidant capacity and quality of tomato fruits under As toxicity. To get a deeper understanding, it is crucial to conduct systematic research on the role of Si-NPs in the absorption and movement of As. This data might aid in creating innovative strategies for the design of nano fertilizers, including Si-NPs, which specifically target the accumulation of As in different plant species.

##### Titanium dioxide (TiO_2_) NPs

Due to their robust physical and chemical stability, low toxicity, corrosion resistance, and affordability, TiO_2_-NPs are gaining increasing acceptance as having considerable potential for environmental applications [57]. TiO_2_-NPs have demonstrated their suitability for ecological remediation due to their potent oxidizing capabilities, excellent resistance to photo-degradation, and selective redox reactions [58]. In most cases of As removal, adsorbents tend to have a higher affinity for As^V^ over As^III^, necessitating pre-oxidation to fully convert As^III^ to As^V^ to utilize the adsorbents’ potential. The inherent photocatalytic activity of TiO_2_-NPs for photo-oxidizing As^III^ to As^V^ offers an additional advantage [57]. Recent studies have investigated the role of three types of TiO_2_-NPs in reducing As^III^ accumulation. TiO_2_-NPs reduced As accumulation by 90% (rutile) and 40% (anatase) compared to the control groups. The accumulation in seedlings decreased by 14% with anatase and 90% with rutile, relative to the control groups. Here, it can be inferred that the primary factors limiting As accumulation, bioavailability, and toxicity are As adsorption on the surface of TiO_2_-NPs and As retention on the Fe plate in the rhizosphere, which is analog to the previously identified mechanisms [58].

Katiyar et al. [59] noted that TiO_2_-NPs counteracted the growth inhibitions and membrane degradation induced by As exposure. Moreover, TiO_2_-NPs reduced the MDA and ROS genesis triggered by As by upregulating antioxidant gene expression. These data indicate that TiO_2_-NPs, particularly those produced by environmentally friendly techniques, efficiently induce the antioxidant capacity of plants, aiding in the mitigation of high ROS levels and the reduction of As stress. Therefore, the use of TiO_2_-NPs generated by green methods seems to be a safe, economical, and eco-friendly strategy, making it a superior alternative to chemically manufactured TiO_2_-NPs. Kiany et al. [18] noted that the utilization of TiO_2_-NPs improves the capacity of rice plants to acclimate to As toxicity. This enhancement is accomplished by upregulating *GSH1* and *PCS* expression, strengthening the process of metal sequestration, and enhancing antioxidant activity. Conversely, different research administered 2000 mg/kg TiO_2_-NPs in conjunction with soil amendments and found little impact on soil properties [60], confirming that TiO_2_-NPs facilitating the detoxification of As species is unlikely to provide substantial concerns in future studies (See Table 3).

**Table 3:** The list of different nanoparticle (NP)s used in different plant species under Pb and Cr toxicity

Heavy metal	NP	NP concentration	Plant species	Details	Ref
Pb	ZV-Fe	1 ~ 3 mg/kg	*Triticum aestivum, Acer velutinum*	Enhanced the growth, yield, and activity of CAT, SOD, urease, and acid phosphatase, declined Pb uptake, MDA, H_2_O_2_, and EL	[147, 149]
	Ag	10 ~ 50 mg L	*Vigna radiate*	Improved growth, biomass, yield, photosynthetic rate, total Chl, water use efficiency, activity of antioxidant enzymes, ionic homeostasis, Decreased Pb uptake, MDA, and ROS content	[150]
	ZV-Ag	0.2 mg/kg	*Moringa oleifera*	Increased growth, germination rate, total flavonoid and phenolic contents, RWC, and photosynthetic pigments, Diminished oxidative stress, and Pb uptake	[232]
	ZnO	5 ~ 50 mg L	*Persicaria hydropiper, Solanum lycopersicum, Basella alba, Triticum aestivum*	Increased growth, germination rate, seedling vigor index, proline, RWC, photosynthetic pigments, phenolics, flavonoids, activation of PAL and antioxidant enzymes, Pb accumulation and translocation	[151, 157, 159, 233]
	SiO_2_	50 ~ 1000 mg/L	*Triticum aestivum, Coriandrum sativum, Ocimum basilicum, Pleioblastus pygmaeus*	Improved growth, proline, phenol, antioxidant capacity, and activity of PAL and antioxidant enzymes, Reduced Pb uptake in root and shoot, Downregulated polyphenol oxidase activity	[153–155, 234]
	MgO	5 ~ 20 mg/L	*Raphanus sativus, Daucus carota*	Increased plant growth, phenolic and flavonoid contents, mineral nutrients, terpenoid, total polyamine content, free radical scavenging activity, and Pb phytoaccumulation, Declined Pb translocation, MDA, and ROS	[156, 157]
	Fe_3_O_4_	200 mg/L	*Basella alba, Coriandrum sativum, Ricinus communis*	Increased seed germination, proline content, nutritional balance, activity of SOD, CAT, and POD, Decreased Pb accumulation and ROS content	[151, 213, 235]
	TiO_2_	5 mg/L	*Lactuca sativa*	Improve growth and gas exchange parameters, Declined Pb uptake, MDA, and ROS	[158]
Cr	ZnO	50 ~ 100 mg/L	*Oryza sativa, Triticum aestivum*,	Enhanced growth, photosynthetic efficiency, nutrient uptake, NO content, activity, and expression of antioxidative enzymes, and AsA-GSH cycle, Reduced Cr uptake, MDA, and ROS content	[197, 198]
	SiO_2_	10 µM	*Pisum sativum, Triticum aestivum, Oryza sativa*	Improve growth, Chl fluorescence, endogenous NO, photosynthetic pigments, and activity of antioxidant enzymes, decreased Cr uptake, and ROS, induced cell cycle at G2/M phase	[185, 187, 188]
	CeO_2_	25–50 mg/L	*Helianthus annuus*	Improved growth, biomass production, photosynthetic pigments, gas exchange parameters, activities of antioxidative enzymes, Reduced oxidative stress, MDA, EL, and Cr uptake	[186]
	Fe_3_O_4_	10–20 mg/L	*Oryza sativa, Triticum aestivum*	Enhanced growth, biomass, yield, photosynthetic activity, micronutrients, gas exchange attributes, and activities of antioxidant enzymes, Reduced oxidative damage, MDA, EL, and the uptake and accumulation of Cr	[189, 190]
	ZV-Fe	5-100 mg/L	*Catharanthus roseus, Cosmos bipinnatus, Gomphrena globose, Impatiens balsamina, Solanum lycoperscium, Helianthus annuus*	Augmented plants’ potential for Cr accumulation without negatively hampering plant growth, Improved germination, hypocotyl and root length, photosynthetic pigments	[183, 236, 237]
	TiO_2_	2.5 mg/L	*Abelmoschus esculentus, Helianthus annuus*	Increased yield, fruit length, height, Chl content, activity of antioxidant enzymes, Reduced Cr accumulation in fruit, root, and stem	[193, 238]
	Cu	25–50 mg/kg	*Triticum aestivum*	Improved growth, biomass, Cr- immobilization in soil, activity of antioxidant enzymes, proline, total phenolics, Declined Cr accumulation in shoot and root, MDA, and H_2_O_2_	[184]

##### Other NPs

There is a scarcity of research documenting the possible functions of other metallic NPs in alleviating the damaging effects of As treatments on higher plants. Nazir et al. [10] demonstrated that CaO-NPs enhanced plant growth in the presence of As toxicity by increasing calcium availability, decreasing As uptake through the downregulation of genes such as *PHT1;1*, *1;3*, *1;4*, and *1;6*, and reinforcing the plant’s capacity to scavenge ROS. Li et al. [61] noted that the coexistence of MnO_2_-NPs with As resulted in a decline in the average As concentration in roots and husks compared to treatments with As alone. This implies that MnO_2_-NPs can promote As^III^ oxidation to As^V^ and hence lessen As bioaccumulation. MgO-NPs effectively buffered oxidative damage by enhancing antioxidant enzyme levels, hence facilitating ROS elimination [16]. Cu-NPs have been proven to have a crucial function in improving food safety and raising the nutritional quality of rice in As-contaminated regions by drastically declining As levels in grains [245]. Chen et al. [62] discovered that multi-walled carbon nanotubes (MWCNTs) had an advantageous role on plant growth and the behavior of As in contaminated soils, resulting in improved shoot length and higher plant dry biomass. At a 1000 mg/kg dosage, MWCNTs impeded the growth of both leaves and roots. In contrast, 500 mg/kg MWCNTs substantially exacerbated HM accumulation, leading to a 32.5% increase in As levels. This increased the micronutrient content, enhanced antioxidant enzyme activity, and improved plant development, all of which reduced the toxicity. Also, applying MWCNTs decreased As’s bio-concentration factor, which was essential in reducing phytotoxicity. There is currently a shortage of research on the possible effects of designed/engineered NPs, such as gold, mercury, graphene, and silver NPs, in reducing the toxicity of both As^III^ and As^V^ in higher plants, and additional research is required at different levels, including physiological, metabolic, biochemical, and molecular factors.

### Deciphering the role of NPs in the alleviation of cd stress

#### The mechanisms of absorption, transport, and sequestration of cd

The plants’ reaction to increased Cd levels demonstrates variety, which may be related to variations in the ability of different plant cultivars and species to transport and absorb Cd and the soil’s physicochemical properties. While the transportation of Cd throughout the plant occurs efficiently via metalloorganic complexes [63], the accessibility of Cd is regulated by variables like Cd content, redox potential, temperature, pH, and the content of other components in the soil. Exuding carboxylase and acidifying the rhizosphere are potential strategies for depositing metals. The process of Cd assimilation by roots involves competition between Cd and other minerals with similar properties for absorption sites. Cd is a suitable substitute for Ca due to its analogous ionic radius, charge, and behavior [64].

Cd initially infiltrates plant roots, causing root system damage and morphological changes. The regulation of Cd transport across root cells` membranes is determined by the differential in electrochemical potential between the apoplasts containing Cd and the cytosol of the root. Even at significantly low concentrations of Cd, the energy supplied by the membrane potential is adequate to promote Cd uptake [65]. The energy necessary for Cd absorption in roots demonstrates biphasic characteristics, consisting of saturable components during low Cd activities and a linear fraction during high Cd activities. In roots, Cd absorption can occur in various forms, including organic forms (like complexes of phytometallophores) or inorganic complexes (such as CdCl^+^, CdCl_2,_ and Cd^2+^SO_4_) [66].

Soil acidity increases the bioavailability of Cd for plants, and the presence of root exudates improves its solubility. Cd is commonly found in its ionic state as Cd^2+^ and may also be detected in the soil solution as Cd-chelates [67]. The symplastic and apoplastic channels are separate ways that have a role in the absorption of metal ions, like Cd [68]. In the apoplast of plant roots, cations are prone to accumulation; this process is impacted by the exchange characteristics of the cell wall and is initially pH-dependent concerning the adsorption of HMs from the soil solution. As the soil pH rises, the functional compounds found in the cell walls of the roots, especially carboxyl groups, undergo a slow deprotonation process [69]. Electrostatic interaction occurs between negatively charged carboxylate compounds and metal cations in the apoplast. This contact is fast and unpredictable and occurs at the root level. Yin et al. [70] noted that this process follows a passive energy pathway; hence, it does not require any energy source. Compared to the apoplastic system, the symplastic pathway is slower and is defined by its need for metabolic activity. The importance of each stage, however, varies for the metal and ion types and concentrations [68]. Cd is transported through the apoplastic channel, which is located on the root cells` cellular membrane [67].

The process by which plants absorb Cd involves a multitude of groups of membrane proteins. Proteins belonging to the ZIP family, including IRT1 and ZRT1, play a part in Cd absorption in plants. Metal transporter ZIPs are unique because they help move metal ions from organelle lumens and extracellular regions into the cytoplasm [71]. One component of Cd absorption in plants is the oligopeptide transporters (OPT), like the yellow-stripe-like (YSL) transporters. They help nicotianamine-metal interactions pass the cell membrane [72]. Other metal transporter groups involved in cation movement are the proton-coupled metal-ion transporters, members of the NRAMP family [73]. It is well-known that divalent cation/metal-transporter-1, which are NRAMP transporters, may transport a variety of HMs, especially Cd [74]. The NRAMP transporters are essential for the uptake of Cd and help translocate Cd from roots to shoots by mediating influx via the endodermal plasma membrane [73]. The guard cells’ plasma membrane Ca^2+^ channels are relatively permeable to Cd. The hindering impact of Cd on epidermal strips provides further evidence for this conclusion. The presence of putative Ca channel inhibitors, including voltage-intensive cation channels (VICCs), and hyperpolarization or depolarization-activated Ca channels, is responsible for this effect. This information suggests that Ca channels may serve as a route for Cd ions to enter guard cells [75].

Cd has significant mobility and assimilation capacity, enabling its uptake by plants via the root. Subsequently, it is transported in its ionic form through transporters and/or by the ascent of sap in shoots, ultimately reaching the vascular bundles, including the phloem and xylem. Cd can infiltrate the xylem via the symplastic route, potentially resulting in a greater Cd concentration in the apoplastic pathway [76]. Cd has been observed to be transmitted from the tracheids or vessel components of the stele to the plant’s shoots. The movement of solutes across the gas pores and extracellular fluid within and between cell walls is promoted by apoplastic pathways [67]. Metal ATPases can expedite the transportation of Cd across cellular membranes, hence playing a pivotal role in the root-to-shoot Cd translocation. The P1B-ATPases are a subset of P-type ATPases that actively transport ions against their electrochemical gradient through cell membranes [77]. P1B-ATPases regulate the flux of metals throughout the cytoplasm, resulting in constant Cd concentrations. Phloem-mediated translocation of Cd into grains might account for the migration of Cd into cereal grains [78]. Phloem is the principal conduit through which Cd is transported into grains. Additionally, Cd is capable of forming complexes with molecules generated by the SH and 13 kD-protein groups present in the phloem sap [69]. At the nodes, there is the xylem-to-phloem Cd transfer, and the transport of phloem Cd along a panicle neck suggests the presence of genetic diversity. This confirms transporters in the nodes and their active role in Cd transport in the phloem towards the grain. However, regardless of the mobility of Cd, concentration in the root is higher than in the shoot [79]. Typically, plants primarily absorb Cd through their roots, with only a small amount being transferred to other parts, like leaves, stems, and reproductive organs. The transportation mechanism exhibits a particular sequence, with the roots showing the highest accumulation, followed by the leaves, fruits, and grains [66]. For instance, the roots of soybean plants retain 98% of the entire quantity of Cd absorption from the soil. The residual portion is subsequently carried to the shoots and other aerial structures by the vascular bundles [80]. Most plants have limited capacity to transport Cd through the xylem, resulting in minimal Cd presence in seeds, fruits, and shoots, demonstrating that Cd is not carried through the phloem [65]. However, certain plant species, like tobacco, possess a higher ability to accumulate metals, leading to more Cd in older leaves [81]. The translocation of Cd into grains and fruits displays variability among crop genotypes. Various factors, including agronomic practices, plant genotypes, environmental factors, and soil features, affect the Cd uptake in different organs [65].

#### NPs-mediated processes for alleviating Cd-induced stress

NPs have been used in several recent research to investigate how to mitigate biotic and abiotic stressors in plants. Regarding the mitigation of Cd stress, however, the effects and mechanisms of NPs remain poorly understood. This chapter is a compilation of the latest study findings and provides an overview of probable pathways that might elucidate the impact of NPs on strengthening Cd tolerance in plants.

##### Decreasing cd uptake in plants

Reducing the absorption and transportation of Cd is one of NPs’ main functions in helping plants cope with Cd stress. As NPs reduce Cd absorption and transport, several studies have shown that their addition can increase plant tolerance to the metal. For instance, ZnO-NPs have been demonstrated to lower Cd levels in maize [82], wheat [19, 83], rice [9], and lettuce [84]. Application of SiNPs by foliar spray led to decreases in Cd levels in grains, roots, and shoots, with the reductions ranging from 20 to 80%, 20–65%, and 16–60%, respectively. Similarly, applying SiNPs to the soil reduced Cd levels in grains, roots, and shoots by 22–82%, 10–60%, and 12–55%, respectively [85]. TiO_2_-NPs are successful in reducing Cd absorption in several plants, including *Coriandrum sativum* [15], bamboo [87], rice [88], and summer savory [89]. The use of CaO-NPs in barley led to a notable reduction in shoot Cd (43–70%) and root (30–40%) of both genotypes [11]. CuONP treatment has also been demonstrated to decrease Cd levels in wheat, rice, and barley plants, concurrent with the plants’ enhanced growth under Cd treatment [90, 91]. One of the main reasons for the decline in Cd absorption might be because NPs have made Cd less available in the soil. The NPs have a large surface area, which makes them very effective in absorbing and aggregating metal ions. This ability allows them to immobilize Cd in the soil [92]. The ideal amounts of Fe_3_O_4_-NPs-modified biochar formed iron dots on the rice root surface, acting as a protective barrier and improving the capacity of the soil to exchange cations. The improvement decreased soil Cd’s effectiveness, resulting in a decline in plant absorption and accumulation [93]. According to Chen et al. [94], applying ZnO-NPs reduced the amount of Cd bioavailable to wheat, decreasing the amount of Cd transferred to the plant. Similarly, SiNPs significantly reduce the biological efficacy of Cd in the soil, which in turn causes a drop in Cd levels in plants [95]. In addition to immediately remedying Cd, the modification of soil pH plays a vital role in the process of Cd passivation. When Fe-NPs are applied to rice plants under Cd stress, the soil pH is significantly raised, which lowers the amount of Cd in the roots and shoots of the rice plants and decreases the bioavailability of Cd in the soil [83]. Combining KH_2_PO_4_ with temperature-activated nano zeolite or nano serpentine can effectively immobilize Cd in polluted soils. This is achieved by reducing Cd’s biological impact through increasing soil pH [96]. During development, plants create physical barriers that hinder Cd absorption into their cells, and NPs can help establish these barriers, mainly when plants are under Cd stress. This can significantly prevent plants from absorbing more Cd [92]. Also, biochar NPs attach to the surface of root tips, forming a protective shell-like structure that functions as a physical barrier. This barrier reduces Cd movement, as demonstrated by Yue et al. [97]. The Fe-NPs led to a 26% elevation in the presence of Fe spots on the rice root system. The Fe spots act as natural barriers, effectively inhibiting Cd absorption by rice [98].

Pore volume, high surface area, and surface-active site qualities are advantageous features of biochar-based nanocomposites. These characteristics enhance the effective adsorption of HM [99]. For example, when biochar is treated with nano-hydroxyapatite, it shows considerable capacity to adsorb Cd. This characteristic facilitates the elimination of Cd from wastewater, alleviates the entry of Cd into the plant’s growth environment, and diminishes the detrimental roles of Cd on plants [100]. ZnO-NPs and biochar showed efficacy in moderating soil Cd’s impact, resulting in a significant decrease in Cd content in alfalfa plants subjected to Cd-induced toxicity [101]. However, there are certain cases in which the utilization of NPs fails to decrease Cd absorption by plants in a beneficial manner. For instance, TiO_2_-NPs (100 and 500 mg/kg) at elevated concentrations augmented the Cd concentration in rice seeds [102]. This can be related to the adsorption of Cd onto TiO_2_-NPs` surface and tandem absorption with TiO_2_-NPs by roots, resulting in a higher uptake by plants. Therefore, in applications, it is vital to choose the suitable form and concentration of NPs carefully, and additional data is required to determine the specific NPs that are most efficient in mitigating the functions of Cd phytotoxicity in plant species. Furthermore, the precise methods by which NPs impact the plant Cd absorption require additional research.

##### NPs-mediated regulation of cd transport in plants

Following root absorption, Cd undergoes both vertical and horizontal transport and redistribution within plants. Consequently, plants develop diverse detoxification mechanisms in reaction to increased Cd levels, including root retention, osmotic adjustment, antioxidation, chelation, and compartmentalization. NPs have been documented to influence the structural components of tissues and plants’ physio-biochemical activities. This regulation by NPs can impact the absorption, distribution, and toxicity of Cd [103].

A way to hinder Cd’s movement from organs is to confine Cd within the root cell wall. Pectin, lignin, hemicellulose, cellulose, and polysaccharides are among the components of the cell wall that provide different aldehyde, hydroxyl, carboxyl, and amino groups that bind and limit Cd [104]. When Si-NP is introduced to plants, the amounts of chelate- and alkali-soluble pectin in the roots rise, and the cell wall’s degree of de-methyl-esterification also rises. Due to these changes, more free carboxyl groups are present, which improves Cd binding and retention in the root cell wall [88]. Cui et al. [105] showed that the strong suppression of Cd absorption in rice root cells treated with SiNPs was due to the enhanced mechanical integrity of the cell walls.

The transportation of Cd within plants encompasses symplastic and apoplastic mechanisms for moving the element into the vascular cylinder. From there, Cd is translocated to the shoots by xylem-loading-mediated processes. At nodes, transport is redirected through intervascular transfer, ultimately leading to Cd transportation into grains. NPs primarily hamper Cd transport in plants via three mechanisms: vacuole sequestration, the formation of an apoplasmic barrier, and the modulation of transporter-related gene expression [103]. NPs have been observed to induce the process of Cd vacuolar sequestration by modulating the expression of ion transporters found on vacuolar membranes. For example, introducing Fe-NP into rice resulted in an upregulation in the *CAX4* expression of the root cells, resulting in Cd accumulation in the root apex and a drop in Cd levels in the leaves [106]. The SiNPs applied to rice suspension cells generated a size-dependent upregulation of the Cd transporter *HMA3* gene expression in vacuoles. This ultimately culminated in Cd toxicity reduction in rice cells [107]. Furthermore, an elevation in the Cd accumulation in cell walls and vacuoles, coupled with a drop in the transport of Cd from roots to shoots, was noted in Se-NP-treated rice, as reported by Xu et al. [108].

During radial transport of Cd from the soil to the root vascular system, Casparian strips and suberin lamellae are thought to function as apoplastic barriers. Previous research has shown that cerium oxide NPs (CeO_2_-NPs) can enhance the formation of Casparian strips and suberin by up-regulating the expression levels of *KCS20, GPAT5, CASP*, and *CYP86A1* [109] and as a result, Cd uptake by the apoplastic pathway was significantly reduced. Rossi et al. [110] and Fox et al. [111] showed that CeO_2_-NPs yielded similar results on soybean and maize. Furthermore, several studies have examined the role of Si-NPs on the induction of “root apoplastic barriers” in plant species exposed to Cd stress. Most showed that SiNPs exhibited better potential than conventional Si in mitigating Cd stress on plants [112, 113].

Certain NPs have demonstrated the ability to modulate the Cd transportation within the plant by altering the expression of transporters. Cui et al. [107] discovered that using SiNPs decreased expression related to Cd transport, specifically the transporter genes responsible for Cd uptake (*Nramp5*) and Cd deposition into grains and phloem (*LCT1*). SiNPs, on the other hand, have been shown to increase the expression of genes involved in Si uptake (*Lsi1*) and Cd transport into vacuoles (*HMA3*). Cui et al. [107] discovered that exposure to SiNPs effectively protects cellular morphology and function from Cd by enhancing Si uptake and lowering Cd uptake. For the duration of the filling stage, 50–100 mol/L foliar SeNPs downregulated the expression of genes encoding transporters involved in the movement of Cd to node I (*LCT1, HMA2*, and *CCX2*) and from node I to grains (*LCT1*, *PCR1*, and *CCX2*). Consequently, the lower expression of transporter genes contributed to reduced Cd accumulation and grain yield in brown rice. Furthermore, studies have demonstrated that SeNPs enhanced the production of Se-binding protein 1 (SBP), inhibiting Cd transportation into rice grains by forming SBP-Cd complexes [114]. Li et al. [115] demonstrated that administering Se-NPs to pepper plants through the root system raised the Cd binding onto lignins. This contributed to activating lignin-associated genes, including *CAD, PAL, COMT*, and *4CL*. Rice plants were subjected to Fe_2_O_3_-NPs, and the results showed that the Fe ions` presence on the root surface had a minimal impact on lowering Cd. Nevertheless, there was a decline in the Cd levels in both the roots and shoots. This decline can be due to the suppression of *Namp5, Cd1, IRT1*, and *IRT2* transporters` gene expression by Fe_2_O_3_-NPs [116]. Ahmed et al. [22] reported similar data about the impact of Fe-NPs on rice plants; they discovered a drop in the expressions of Cd transporter genes, such as *HMA2, HMA3*, and *LCT1* (See Table 4).

**Table 4:** The list of different nanoparticle (NP)s used in different plant species under Cu, Al, Ni, and Zn toxicity

Heavy metal	NP	NP concentration	Plant species	Details	Ref
Cu	S	300 mg/L	*Brassica napus*	Increased growth, height, Chl content, nutrient uptake, activity of antioxidant enzymes, declined Cu uptake and MDA in root and shoot	[201]
	C	5 ~ 200 mg/L	*Zea mays*	Increased germination, seedling length, fresh biomass, activity of antioxidant enzymes, and Cu accumulation, decreased germination time	[202]
	Fe_2_O_3_	2 g/kg	*Ricinus communis*	Enhanced growth and biomass	[213]
	Fe_3_O_4_	2000 mg/L	*Triticum aestivum*	Improved growth, biomass, and activity of SOD and CAT, decreased Cu uptake and MDA	[204]
	PSI	5 ~ 160 mg/L	*Zea mays*	Improved seed germination, seed imbibition, antioxidant enzyme activity, and Cu uptake	[205]
	ZnO	50 mg/kg	*Solanum lycopersicum*	Increased growth and biomass, Decreased H_2_O_2_ contents, EL, MDA content, Decreased Cu uptake	[239]
	CNT	5 ~ 200 mg/L	*Zea mays*	Enhanced germination, seedling length, fresh biomass, activity of SOD, CAT, and POD, and Cu accumulation, Decreased germination time	[202]
Al	SiO_2_	3–5%, 4 mg/kg	*Cicer arietinum, Zea mays*	Enhanced seedling growth, germination percentage, photosynthetic pigments, gas exchange-related parameters, membrane stability index, organic acid, and activity/expression of SOD, CAT, and APX, Declined MDA, H_2_O_2_, and superoxide radicals levels, activity of glycolate oxidase, hydroxy pyruvate reductase, and NADPH oxidase	[208, 209]
	TiO_2_	5 mg/L	*Lactuca sativa*	Improved germination rate, seedling length, water content, anthocyanins and water content, photosynthesis related-parameters, and efficiency of photosystem II	[158]
Ni	SiO_2_	2.5-5 mM/L	*Phaseolus vulgaris*	Enhanced growth, biomass, and antioxidative defense system, decreased H_2_O_2_ contents, EL, MDA content, and Ni concentration in the leaves	[212]
Zn	Fe_3_O_4_	2000 mg/L	*Triticum aestivum*	Improved growth, biomass, and activity Mariz of SOD and CAT enzymes, decreased Cu uptake, ROS, and MDA	[204]
	Fe_2_O_3_	2 g/kg	*Ricinus communis*	Enhanced growth and biomass	[213]

##### Detoxification of cd through NPs-facilitated physiological and metabolic processes

The potential consequence of Cd entering plant cells is that it could vie for channel transporters with vital nutrients, leading to subsequent nutritional deficiency in plants. Plant Cd absorption, accumulation, transport, and toxicity have all been reported to be inhibited by NPs’ optimization of mineral nutrients [92]. For example, prior research has shown that FeO- and CuO-NPs may significantly reduce the amount of Cd that accumulates in plants by promoting the translocation of vital elements, including K, Mg, Fe, and Zn [90, 117]. Applying ZnO-NPs to plants has been discovered to improve Zn nutrition, protein levels, and the activities of carbonic anhydrase and nitrate reductase, especially when plants are under stress caused by Cd. Kareem et al. [101] have demonstrated the substantial influence of these effects in reducing Cd accumulation and its detrimental impacts on plants. According to Ogunkunle et al. [118], applying TiO_2_-NPs to cowpea plants has also been demonstrated to improve the absorption of macro- and micronutrients, including P, N, Co, K, Mn, Zn, Fe, Mg, and Ca. Likewise, it has been discovered that exposing tomato plants’ roots to Fe_3_O_4_ NPs enhances the absorption of these vital elements [119]. The NPs derived from astaxanthin (AstNP) to wheat plants applied hydroponically have also demonstrated enhanced assimilation of P, N, K, Zn, Mn, Co, Fe, Mg, and Ca [120]. The improved mechanisms of nutrient assimilation and transport in plants not only facilitate the transportation of Cd through complete transporters but also boost the plants’ resistance, leading to a reduction in Cd buildup and mitigating its harmful effects.

Most of the NPs that have been established in several studies to have positive effects in reducing Cd toxicity can also strengthen the defense system in plants. This functions as a means of protection against oxidative damage caused by Cd. Prominent instances are ZnO-NPs [9, 82, 84, 121, 122], Fe-NPs [117, 119, 123, 124], TiO_2_-NPs [15, 122], SiO_2_-NPs [88, 117, 125], CaO-NPs [11], Se-NPs [86, 126, 127], Ag-NPs [128], and CuO-NPs [90, 91]. Cowpea plants’ roots and leaves exhibited increased CAT and APX activities and decreased MDA following the application of TiO_2_-NPs [118]. Using Si-NPs and Fe-NPs on *Phaseolus vulgaris* subjected to Cd pollution raised the SOD and CAT while lowering MDA content and electrolyte leakage (EL), according to Koleva et al. [117]. Rice plants under the Cd effect increased SOD, POD, CAT, and APX activities when ZnO-NPs were introduced, as shown by Ghouri et al. [129]. This increase in enzymatic activity resulted in a decline in the levels of H_2_O_2_, EL, and MDA.

Additionally, research has shown that NPs can mitigate Cd stress on plants through the enhancement of the non-enzymatic antioxidant system. When *Azolla filiculoides* were exposed to Cd-induced stress, the proline content rose 3.9 times when TiO_2_-NPs were applied [130]. The results were similar for both Fe-NPs and SiNPs, according to Koleva et al. [117], who noticed an elevation in the proline content of *Phaseolus vulgaris*. According to Nazir et al. [11], the presence of CaO-NPs has been found to enhance the activity of antioxidant enzymes and elevate the levels of GSH and ASA in barley plants subjected to Cd-induced stress. Applying Se-NPs in the presence of Cd and other HM stress conditions resulted in a considerable elevation of ASA and GSH in *Brassica chinensis* [131]. On the other hand, when bamboo plants were stressed by Cd, TiO_2_-NPs significantly increased levels of non-enzymatic antioxidants such as tocopherols, flavonols, and total phenolics [87]. The application of ZnO-NP resulted in an elevation of CAT, SOD, and POD activity in *Cucumis melo* subjected to Cd-induced stress, as well as an augmentation of flavonoids and phenolics [132]. Reduced generation and accumulation of H_2_O_2_ were the benefits of using Se-NPs. This reduction was achieved by downregulating the expression of specific enzymes, namely NADPH oxidases (*RBOHC*, *RBOHD1*, and *RBOHF1*) and ethanoic acid oxidase. Consequently, the impact of SeNPs on Cd-induced membrane lipid degradation in *Brassica napus* was shown to be mitigated [127].

The Si-NPs in various utilizations, including root exposure, seed priming, and foliar spray, have been found to enhance the photosynthetic activity of rice [88], *Phaseolus vulgaris* [117], and bitter gourd [133] as a means to mitigate the adverse effects of Cd stress. The SeNPs at ideal doses have been found to positively affect the expression of Lhcb1, psbA, and RbcL proteins, as well as enhance Rubisco activities and chlorophyll levels under Cd-induced stress [114]. The promotion of photosynthetic parameters can be observed in plants treated with ZnO-NPs under Cd stress through many routes, including leaf [9], root [101], and seed [83]. Furthermore, Kareem et al. [101] conducted a study that revealed that the presence of ZnO-NPs within the mesophyll cells of alfalfa leaves resulted in an improvement in the cellular ultrastructure severely distorted by Cd toxicity. This improvement was characterized by the restoration of a regular cell shape with a well-defined cell membrane and cell wall, the presence of properly formed ellipsoidal chloroplasts with reduced starch granules, increased ribosome content, and the orderly arrangement of grana lamellae and chloroplast thylakoids.

Studies have investigated the functions of NPs during Cd stress on plant metabolomics. Li et al. [115] found that pepper plants treated with Se-NPs had significantly higher amounts of salicylic acid, capsaicinoid, jasmonic acid, and chemicals associated with the proline pathway in the leaves. The production of antioxidants and secondary metabolites is known to rely heavily on these substances. Therefore, pepper plants exposed to Cd stress and simultaneously treated with Se-NPs had increased concentrations of dihydrocapsaicin, nordihydrocapsaicin, and additional amino acids in the roots and fruits. The concentration of core and secondary metabolites in the treated plants also increased. Therefore, pepper plants exposed to SeNPs had a far better ability to withstand Cd stress, allowing them to produce superior fruits [115]. Applying ZnO-NPs to the seeds of two fragrant rice cultivars in the presence of 100 mg/L Cd stress increased enzymatic and non-enzymatic antioxidants and seedling development. Numerous metabolic routes could potentially be crucial to how rice reacts to ZnO-NPs and Cd combined treatments. These processes include the metabolism of aspartate, taurine, hypotaurine, glutamate, and alanine and the formation of phenylpropanoid [134].

### Mechanistic insights on nanoparticles-mediated lead stress regulation

#### Sources of pollution and absorption of Pb and its toxic effects on plants

**Fig. 5:**
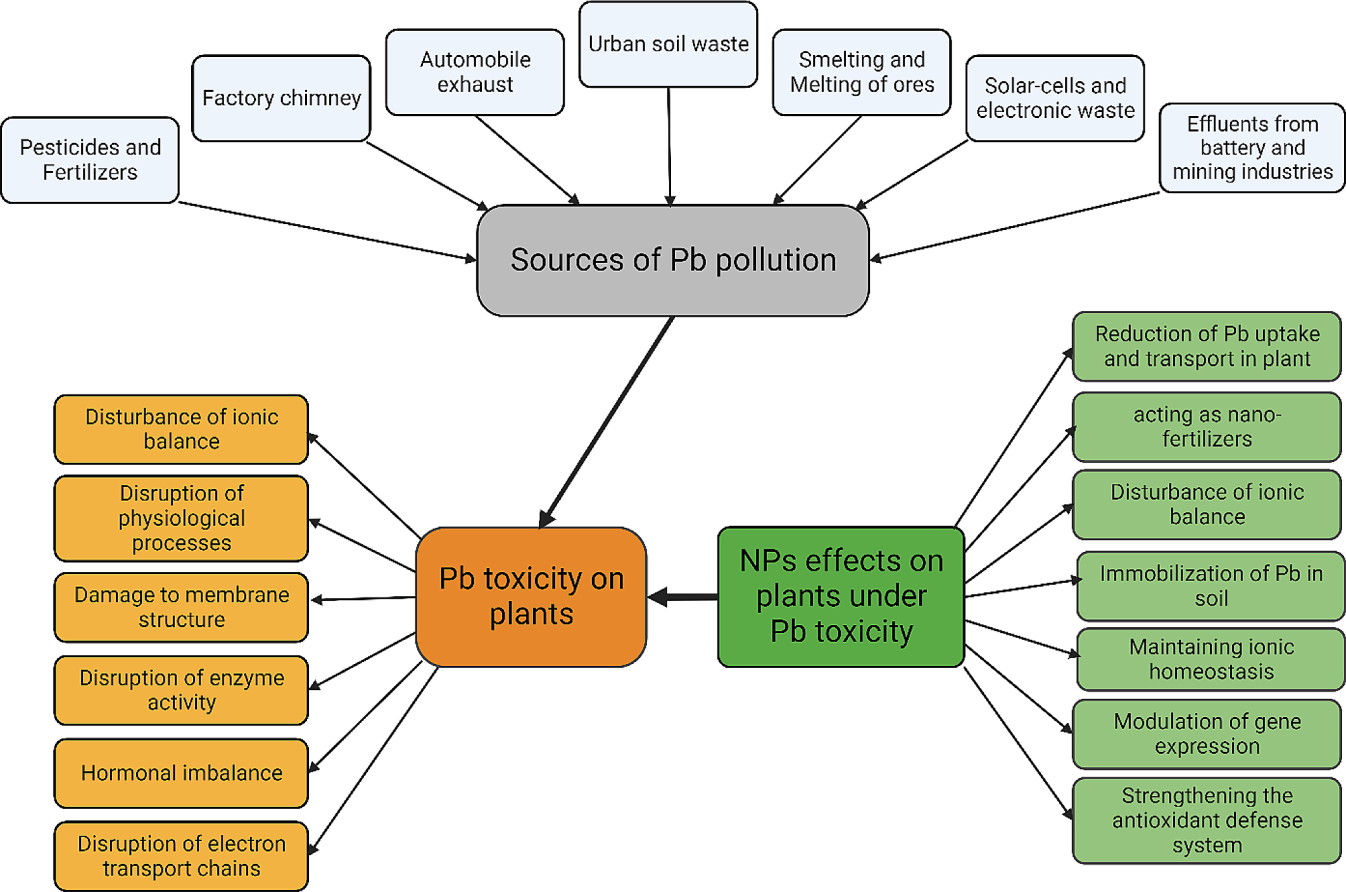
The primary sources of lead (Pb) contamination in the environment, the effects of Pb toxicity on plants, and the impact of nanoparticle (NP) application in reducing Pb-induced toxicity

Lead (Pb) is a prevalent HM pollutant found in soils, posing significant risks to plant life. The pollution of the ecosystem is attributed to the release of contaminants by transport vehicles and refining industries. The amount of Pb contamination caused by HMs is around 10%. Pb is used in various industries, like building, paints, petrochemical refineries, gasoline alkyl addition, lead batteries, and cable coating [135]. Significant emissions of Pb are related to anthropogenic activities, such as electroplating, metal smelting, mining operations, gas exhaust, or energy and fuel-producing power lines (Fig. 5). Gupta et al. [136] detected fluctuations in the isotopic nature of Pb over different time intervals using lichens as a bioindicator. Soils affected by Pb contamination typically exhibit Pb levels ranging from 400 to 800 mg/kg, whereas in industrialized regions, this can reach 1000 mg/kg [137]. Pb in soil can be present in several states, such as freely occurring metal ions attached to inorganic components like SO_4_^2–^, CO_3_^2–^, and HCO_3_^–^, or combined with organic substances such as humic acids, fulvic acids, and amino acids. In addition, Pb may also be adsorbed onto the surfaces of particles, including organic materials, biological substances, and Fe-oxides [135]. Pb in the soil may cause many ionic bonds to form. This is explained by Pb’s classification as a weak Lewis acid, which has a strong covalent bond [138]. Several mechanisms, including chemical ones like reduction or oxidation, chelation aided by metal oxides and organic matter, cation adsorption on the exchange complex, and vegetation-mediated cycling, all impact Pb distribution in soil. Pb’s strong affinity for organic and colloidal molecules increases its solubility in soil and makes it available for plant absorption [139]. The acidity level of soil plays a key role in influencing its ability to retain Pb and plants grown in soil have been reported to absorb more Pb when cultivated in acidity compared to alkaline environments [138].

The plants obtain the absorbed free-Pb ions capillary or through ambient air intake during cellular respiration. Pb enters the plant’s biological system after being directly absorbed by the surrounding environment. Aside from necessary components, such as divalent free-Pb cations, the polluted soil also harbors these cations, which plants assimilate through passive processes. The Pb ions that have been adsorbed are then transferred via the xylem vessels. HM translocation within plants occurs through the xylem vessels, which are transported in an upward flow alongside other dissolved nutrients. The HM is finally released into the endodermis [140], and the large surface area of leaves enables the absorption of metal ions from contaminated air through the stomata and cuticle, leading to leaf chlorosis. This process happens concurrently, expanding towards the endodermis area and establishing connections with the cell walls and membranes [138].

According to the criteria set by the Agency for Hazardous Substances and Disease Registry, four well-known HMs, specifically Hg, Pb, Cd, and As, are commonly found in the environment and are considered highly toxic/have hazardous properties because they can be easily absorbed by plants and animals [141]. Pb is widely dispersed across natural sources and is considered one of the most uniformly dispersed trace metals. Toxic trace metals are present in several forms. Plants have been observed to uptake Pb in the rhizosphere, especially in urban areas where soil contamination occurs as a result of vehicular emissions, as well as in agricultural fields where fertilizers containing HM elements are applied. Pb^2+^, being non-biodegradable, has been found to negatively impact plants and soil, including cation exchange capacity, pH, and organic carbon [138]. According to the standards established by the World Health Organization, the acceptable limit for Pb concentration in soil is 85 ppm, while for plants, it is 2 ppm. Ullah et al. [142] presented results about the average concentrations of Pb in soil and plants, which were found to vary from 2 to 300 and 0.1 to 5.0 mg/kg, respectively, which might impact soil characteristics and agricultural yield. Even a small quantity of Pb can interfere with several biological functions, such as the efficiency of water absorption. This disturbance is marked by signs such as the emergence of brown abbreviated roots, hindered photosynthesis, withering of mature leaves, and stunted growth. These impacts result in delayed plant development [135].

Pb has been shown to interfere with essential plant processes like chlorophyll production, cell division, root extension, transpiration, germination, and seedling growth [140]. Many metabolic enzymes have active groups that can interact with each other, like the phosphate groups of ADP or ATP. Important ions can also be switched out to change the permeability of the cell membrane. Such modifications can potentially induce toxicity in plants [137]. Because Pb is hazardous, it stops making ATP and causes DNA damage and lipid breakdown by producing excessive ROS. The method by which Pb binds to parts of the cell wall or membrane substantially changes the cell wall’s flexibility. Making these changes starts lipid peroxidation, which lowers the membrane potential [143]. According to the research conducted by Antosiewicz and Wierzbicka [144], Pb exposure caused microtubular architecture to dislocate and cell wall flexibility to be disrupted and can work as an antimitotic drug.

Pb stress in plants hampers the regular operation of enzymes that participate in diverse metabolic processes since enzymes are the main target of toxicity. Enzymatic inhibition of up to 50% has been found when Pb is present at concentrations ranging from 10^− 5^ to 2.10^− 4^ M. The extent of this inhibition is measured by the inactivation constant [145], which is higher for Pb than other HMs [146]. The literature suggests that Pb inhibits enzymes in two ways. While demonstrating enzymatic inhibition mediated by Pb may pose challenges, it is evident that other cations with comparable affinity for functional groups of proteins exhibit such inhibition. In the initial process, it is well-established that Pb engages in direct interaction with the ligand group present in enzymes, such as the –SH group. This contact inhibits enzyme activity by masking the catalytically active groups. Furthermore, the obstruction of the -COOH group by Pb has also been evidenced [140]. In the second pathway, Pb may affect vital mineral absorption, such as Fe, Zn, or Mg, which are required for metalloenzymatic activity [135]. The divalent cation possesses the capacity to substitute itself with another cation, leading to the inactivation of the enzyme that is specifically linked to δ-aminolevulinate dehydratase, a crucial enzyme involved in chlorophyll production [138].

#### Mechanism of action of NPs to mitigate pb stress

##### Impact of NPs application on pb uptake and transport in plants under pb stress

Pollution-related variables, including plant species, soil physicochemical properties, and Pb content, influence Pb distribution, accumulation, and transfer in soil and plant sections. NPs have been shown to impede the absorption of Pb by plants. According to Noman et al. [147], applying Fe-NPs decreased Pb absorption and accumulation in wheat plant roots, shoots, and grains by 29, 20, and 32%, respectively. The residual fraction exhibited an increase, while the exchangeable fractions decreased following the administration of Fe-NPs to the soil. The application of Fe-NPs may lead to a reduction in the mobility and bioavailability of Pb in the soil. The change in soil redox potential and the resulting formation of sulfide precipitates can be ascribed to this process [148]. The soil pH has a significant impact on the formation of exchangeable Pb species, with more significant levels of stable fractions observed under alkaline pH conditions. A higher pH and more abundant supply of Fe are obtained via applying Fe-NPs to the soil. These modifications enhance the hydrolysis of Pb in the soil and facilitate the formation of precipitates. As a result, the soil contains high amounts of stable Pb. Tafazoli et al. [149] suggest that using Fe-NPs at a concentration of 3 mg/kg might effectively reduce the bioaccessibility of Pb in polluted soil and promote the development of maple seedlings. In two varieties of *Vigna radiata* exposed to Pb toxicity, Chen et al. [150] demonstrated that Ag-NPs at 25 and 50 mg/L effectively reduced the absorption of Pb in the roots and their transfer to the leaves, thereby protecting the leaves and the photosynthesis process against Pb toxicity.

Under HM toxicity, the application of NPs for seed priming has proven effective in promoting germination and plant growth. Gupta et al. [151], for instance, showed that using ZnO-NPs (200 mg/L) and Fe-NPs (200 mg/L) as priming agents on *Bacella alba* seeds led to a major decrease in Pb content, with 34% and 33% reductions, respectively, resulting in improved germination and growth of seedlings in the face of Pb-induced stress. On the other hand, employing ZnO-NPs caused increased Pb deposition in different *Persicaria hydropiper* tissues. Utilizing ZnO-NPs improved the phytoremediation efficacy of HMs inside these marsh plants [152]. Therefore, NPs can provide a wide range of results regarding Pb absorption and transportation in plants exposed to Pb toxicity due to genetic variances among plant species as well as differences in the nature and physicochemical properties of NPs.

The use of Si-NPs has shown considerable promise in reducing the absorption of Pb in plants exposed to Pb-induced stress. Pb accumulation inside the roots and leaves of basil plants exposed to Pb toxicity was shown to be significantly reduced upon the application of green-synthesized Si-NPs [153]. The authors hypothesized that the decrease in Pb absorption and accumulation might be related to the influence of Si-NPs on suppressing the *HAM2* gene, which is responsible for Pb transportation, together with the simultaneous increase in soil pH. Rahman et al. [154] and Fatemi et al. [155] have also reported identical findings on reducing Pb toxicity in wheat and coriander using Si-NPs. These offer additional data that supports the effectiveness of Si-NPs in reducing Pb absorption and accumulation in plants grown in locations polluted with Pb.

Prior research has shown that applying MgO-NPs along with Pb prompted a significant reduction in the absorption and accumulation of Pb in different plant tissues. Applying MgO-NPs at 5 mmol/L significantly affected the Pb transfer factor, which in turn reduced the Pb amount that accumulated in the roots and shoots of *Daucus carota* plants when Pb was at toxic levels, as shown by Faiz et al. [156]. Improvements in nutritional absorption and preservation of ionic equilibrium were shown to be linked to the decrease in Pb accumulation. According to another work, using green-synthesized MgO-NPs and Pb together significantly reduced the Pb amount taken up by the roots and its movement to the plant’s upper parts [157]. Mariz-Ponte et al. [158] investigated the effect of TiO_2_-NPs on lettuce plants subjected to Al and Pb toxicity. The data showed that having TiO_2_-NPs in the plant prevented the accumulation of Al. In contrast, there was a significant increase in Pb accumulation in the presence of TiO_2_-NPs, and plants could handle Pb toxicity better when TiO_2_-NPs were present. This suggests that TiO_2_-NPs may help lettuce manage Pb toxicity by changing how the plant absorbs and makes Pb available, as well as Pb movement and storage. The large surface area and strong sorption ability of NPs are responsible for the decreased absorption and translocation of Pb by plants. Alterations in gene expression and the induction of structural modifications are also proposed mechanisms by which NPs can inhibit the translocation of Pb from roots to shoots [153, 156].

##### Effect of NPs application on photosynthetic parameters and ionic homeostasis of plants under pb stress

Plant physiological systems such as transpiration rate, stomata conductance (gs), photosynthesis rate, and water consumption efficiency become disrupted under Pb stress. It also drastically lowers the amount of photosynthetic pigments. Collin et al. [135] showed that Pb has a variety of effects on the photosynthetic process. The morphology of chloroplasts and metabolic pathways undergo the most significant shifts. Higher dosages have an additional impact on photochemical efficiency. On the other hand, NPs might improve photosynthesis in plants by enhancing the effectiveness of the light-harvesting complex (LHC). The rate of photosynthesis may be altered by regulating several genes coding enzymes, including carbonic anhydrase, phosphoenolpyruvate (PEP) carboxylase, and RuBisCO [135].

Chlorophyll a, b, carotenoids, and total pigment amount dropped in wheat plants exposed to Pb concentrations of 25 and 50 µM due to the negative effects on the photosynthetic system. Sodium silicate and SiO_2_-NPs, on the other hand, significantly protected the photosynthetic apparatus and improved the pigments in wheat plants exposed to lead stress. Compared to the silicon amendment form that does not contain NPs, results show that SiO_2_-NPs were more effective at protecting the photosynthetic apparatus [154]. ZnO-NPs at concentrations ranging from 5 to 20 mg/L were shown by Hussain et al. [152] to improve carotenoids and chlorophyll in Pb-stressed *Persicaria hydropiper* and to lessen the adverse effects of Pb toxicity on the photosynthetic machinery. The protective impact of ZnO-NPs on photosynthetic pigments may be explained by that ZnO-NPs increase the plant’s total phenolic and flavonoids, which in turn grows the plant’s antioxidant capacity. For instance, when Si-NPs were applied as a 1.5 mM foliar spray, coriander plants exposed to 500 mg/kg of Pb-contaminated soil showed higher levels of photosynthetic pigments [155]. Since Si is a signaling molecule and strengthens the plant’s antioxidant defense systems, it may help buffer the effects of ROS and preserve membrane integrity in the presence of Pb toxicity. This provides a potential explanation for the positive impact of Si-NPs. Utilizing TiO-NPs in the presence of a 5 ppm Pb concentration caused lettuce plants to exhibit improved gas exchange characteristics along with increased levels of carotenoid content, chlorophyll a, chlorophyll b, and the a/b ratio. AgNPs at 10 to 50 mg/L were also shown to improve the photosynthetic apparatus’s performance and increase the photosynthetic pigment levels in *Vigna radiata*. The benefit was attributed to the capacity of AgNPs to improve ion homeostasis and uphold an appropriate antioxidant equilibrium in the plants [150]. The increased rate of water photolysis, along with the increased ribulose-1, 5-bisphosphate oxygenase/carboxylase activity and the electron transport chain, are considered to be responsible for the elevation in photosynthetic rate that has been reported after exposure to NPs. Azim et al. [159] demonstrated that the use of ZnO-NPs, created using *Vernonia cinerea* leaf extract, led to the recovery of photosynthetic pigments in tomato plants exposed to Pb-induced stress. The effects of ZnO-NPs on the protection of photosynthetic pigments during conditions of Pb toxicity may be due to the role of Zn in the development of chlorophyll. This is accomplished by conserving the sulfhydryl groups of the chlorophyll molecule. Moreover, Zn contributes to the repair of photosystem II by aiding in the regeneration of the damaged D1 protein [160]. Prior research has shown that the use of MgO-NPs has an uplifting effect on the chlorophyll levels and net photosynthesis ratio of *Daucus carota* plants subjected to Pb. The enhancement can be attributed to the higher accessibility of crucial nutrients, particularly Fe and Mg, which play a role in chlorophyll biogenesis when MgO-NPs are present in Pb toxicity conditions [156]. Consequently, Pb stress dramatically reduces a wide range of photosynthetic indicators, and NPs have been shown to improve these parameters significantly. This improvement can be linked to the plants’ reduced uptake and mobility of Pb, activating enzymes/antioxidants, and reducing oxidative stress. Additional research on the implications of NPs on the transcript expression and function of enzymes related to photosynthesis can provide a more thorough comprehension of NPs` role in the photosynthetic process under the pressure of HM toxicity.

Pb impedes the uptake of nutritional elements due to its structural analogy to important ions. Therefore, there is a decline in the formation of new roots and an obstacle in their ability to penetrate nutrients. Pb displaces nutrients from their physiologically relevant binding sites, reducing the translocation of critical components. Furthermore, Pb induces a decrease in the presence of H^+^-ATPase on the plasma membrane and promotes the creation of insoluble substances. In addition, Pb forms participating bonds with carrier channels, which further hinders the absorption of nutrients [135]. Pb’s influence on nutrient absorption and translocation processes demonstrates diversity among several plant species [156]. NPs under Pb stress enhance the absorption of nutrient elements in plants. Tafazoli et al. [149] examined the impact of Fe-NPs on *Acer velutinum* in the event of Pb and Cd toxicity. Their findings demonstrated that Fe-NPs (1, 2, and 3 mg/kg) resulted in a growth of the levels of P, N, and K elements in the leaves. The rise in nutrient content in the leaves may be connected to the beneficial impact of Fe-NPs on root development and enhanced endocytosis of root cells. In addition, they mentioned that Fe-NPs decrease the bioavailability of HMs in the rhizosphere, hence diminishing the competition between HMs and nutrients for plant absorption. Faiz et al. [156] established the effectiveness of integrating MgO-NPs into the growing process of *Daucus carota*. MgO-NPs led to increased absorption and accumulation of an assortment of crucial elements, such as Cu, Zn, K, Ca, P, Mg, N, S, and Mn, in the roots and leaves of the carrot plants, especially when exposed to Pb toxicity. The increased absorption of elements observed when MgO-NPs are applied might be ascribed to strengthening antioxidant defenses and protecting cellular membranes against damage caused by oxidative stress. According to a different study, when two mung bean genotypes were subjected to Pb toxicity, AgNPs raised the Ca, P, and K levels in their roots and leaves. Also, the application of CaO-NPs led to increased Ca and K levels in *Abelmoschus esculentus* when subjected to Pb stress [161]. The studies above illustrate the ability of NPs to improve the absorption of nutrients in the context of stress caused by Pb. The possible effect of NP induction on improving nutrient absorption may be associated with the stimulation of root development by reducing the uptake and movement of lead from the soil to different parts of the plant, promoting photosynthetic performance, and reducing oxidative stress; however, the fundamental mechanism responsible for this must be clarified.

##### Impact of NPs application on ROS mitigation in Pb-stressed plants

Plant oxidation can result from redox-active metals like Pb producing ROS through the Haber-Weiss and Fenton processes [135]. Studies have demonstrated that NPs efficiently reduce ROS production and the resulting oxidative damage in plants exposed to Pb. Hussain et al. [152] investigated the effects of different ZnO-NPs concentrations (ranging from 5 to 20 mg/L) on *Persicaria hydropiper* plants exposed to Pb toxicity of 50 mg/L, and ZnO-NPs led to boosted activity of APX, POD, PAL, and SOD enzymes, along with high accumulation of flavonoids and total phenol in the root, leaf, and stems. The changes were linked to improving the plant’s antioxidant activity in the presence of Pb toxic exposure. Three kinds of NPs; Si-NPs, ZnO-NPs, and Se-NPs, were investigated in another study for their effects on sage plants that were exposed to Pb toxicity and compared to plants that were subjected to Pb stress only; the results showed that the administration of all three NPs significantly reduced the amounts of MDA and EL in the plant leaves. Utilizing ZnO-NPs showed the greatest decrease in MDA and EL levels [162]. The CAT, SOD, GR, and APX were observed to increase by CaO-NPs, according to Raza et al. [161]. This decreased the H_2_O_2_, MDA, and EL, alleviating the oxidative stress caused by Pb toxicity in Abelmoschus esculentus and stimulating plant growth. Also, Fatemi et al. [155] discovered that adding Si-NPs to cilantro plants stressed by Pb improved antioxidant enzymes and decreased the MDA amount in the cells. Furthermore, the MgO-NPs, either alone or in combination with thidiazuron, enhanced the ability of radish plants exposed to Pb-induced stress to remove free radicals [157]. The increase in the antioxidant activity caused by the treatment with MgO-NPs may be due to the enhanced synthesis of secondary metabolites stimulated by MgO-NPs application in plants. Hence, the observed decline in oxidative damage in plants subjected to Pb stress could be ascribed to decreased Pb uptake and translocation from the soil to plant organs, along with better photosynthesis and the stimulation of defense mechanisms upon NPs treatment.

### Reprogramming of cr toxicity under the influence of nanoparticles

#### Sources of cr contamination in soil and absorption and transport of Cr in plants

**Fig. 6:**
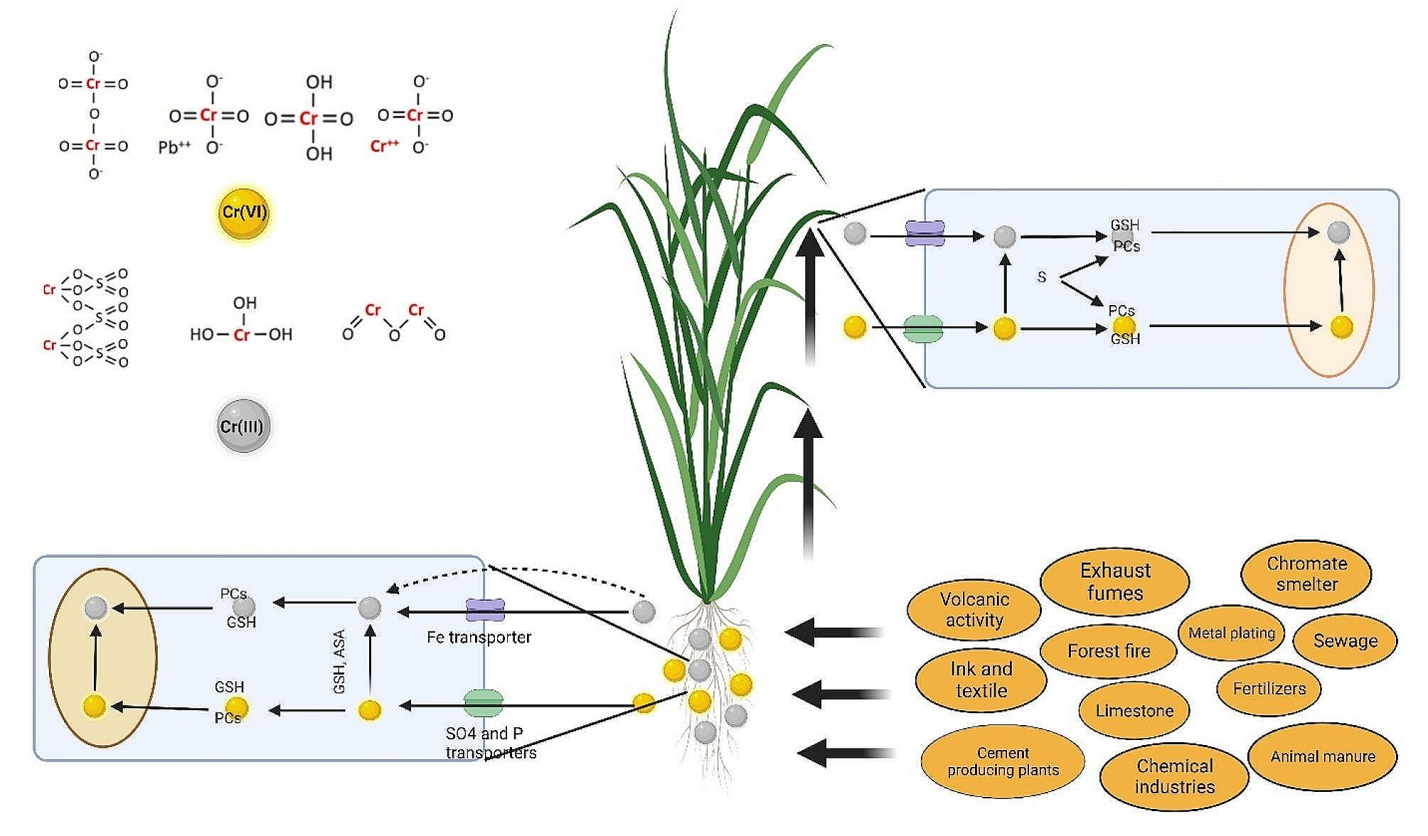
The sources and chemical structures of current Cr(VI) and Cr(III) species in the soils and a diagram illustrating plants’ Cr absorption, movement, and elimination processes

Cr naturally exists in soil, gases, volcanic dust, rocks, animals, and plants. However, anthropogenic activities may account for the vast majority of atmospheric Cr emissions. Its derivatives also possess a wide array of applications in industrial sectors due to their favorable traits, such as hardness and corrosion resistance [163]. The total Cr toxicity in the ecosystem is impacted by both natural and anthropogenic sources. Cr is derived from steel production, metal plating, leather tanning, textile painting and dyeing, metallurgical plating, electroplating, cement manufacturing, alloying, ceramic glazes, pigments, magnetic tapes, refractory bricks, power plants, and other industrial processes [164]. A substantial volume of wastewater with high levels of Cr, generated by several sectors, is released into agricultural fields in developing countries. The leather market accounts for 40% of the overall industrial pollution attributed to Cr [163]. Cr is naturally present in several sources, including volcanic dust, rocks, soil, gases, animal, and plant cells, and it is frequently linked to primary rock-derived phases and highly crystalline iron oxides. Chromite, having the chemical formula FeCr_2_O_4_, is a naturally-occurring compound of Cr found in ultramafic rocks and serpentine. Vauquelinite, crocoite, tarapacaite, and bentorite are among the minerals that include Cr [85]. Furthermore, significant quantities of Cr are released into aquatic environments through the route of natural leaching from rocks and soils. Cr may exist in multiple valence states, ranging from 0 to VI. In contrast to other forms, Cr(VI) and Cr(III) are more stable and prevalent. Cr(III) typically appears as cations with a positive charge, while Cr(VI) exists as chromate (CrO_4_^2−^) or dichromate (Cr_2_O_7_^2−^) oxyanions coupled with oxygen [163, 165]. Cr(VI) exhibits high solubility in soil, is highly harmful to organisms, and is capable of causing carcinogenic, mutagenic, and teratogenic reactions; thus, it poses a possible threat to human health [166]. Cr(VI) compounds are commonly found in environments characterized by abundant oxygen and a pH ranging from alkaline to neutral [165]. When immersed in an acidic solution, it demonstrates a significantly elevated positive redox potential of 1.38 V, implying an extensive ability for oxidation [167]. The primary Cr forms can undergo a series of alterations, moving from one form to another, due to physicochemical processes. During the chemical transformation of Cr(VI) and Cr(III), intermediate states Cr(IV) and Cr(V) are common, and these states are known for their significant instability [168]. In reduced soil conditions, Cr endures precipitation and immobilization. Moreover, Cr(III) is converted from its toxic form, Cr(VI), into a less hazardous form [165]. Xiao et al. [169] identified a direct relationship between the decrease in Cr(VI) and several metrics like the bacterial diversity index of total community, organic matter, Fe(II) concentration, and clay percentage in soils. In contrast, a negative association emerged between the decrease in Cr(VI) and the quantity of Mn that may be reduced in soils. In the soil, metals like Cr can be in different oxidation states, and the soil redox potential is an important factor that changes their biological behavior [170]. The pH of the soil is also a critical factor in influencing the chemical form of Cr. For instance, raising the soil pH causes a decrease in the presence of positive charges, thereby resulting in a decline in the absorption of Cr [171]. On the other hand, Cr(III) has extremely low solubility at a pH of 5.5. It exhibits near-total precipitation when the pH exceeds 5.5, indicating superior durability in soil environments. Cr(VI) has significant instability and may be mobilized under both alkaline and acidic soil pH conditions [172]. Cr(VI) bio-reduction may be directly observed by microbial metabolism under aerobic circumstances, as demonstrated by Qian et al. [173]. Various bacteria can reduce Cr(VI) levels in both anaerobic and oxygen-rich environments [166]. Chromate reductases, including YieF, NemA, ChrR, and LpDH, aid in the transformation of Cr(VI) into Cr(III) in bacteria that resist Cr. The conversion occurs by electron transfer from electron donors (NAD(P)H) to Cr(VI), triggering ROS production [174] (See Fig. 6).

Plants primarily acquire Cr through specialized transporters that facilitate the absorption of ions necessary for metabolic activities. They can assimilate various forms of Cr; however, the specific process through which they accomplish the process remains unidentified. Plant absorption, accumulation, and movement exhibit variations due to Cr`s specific chemical forms, active in metal speciation, which ultimately govern its detrimental effect. Sulfate, a crucial anion transporter, has been discovered to be active in transporting Cr. Studies have demonstrated that Cr competes with S, P, and Fe for carrier binding during transit [175]. Thanks to comparable structures, plants effectively absorb Cr(VI) using phosphate or sulfate transporters [176]. The SULTR gene family, commonly known as H^+^/SO_4_^2−^ transporters, has been identified in every photosynthetic species studied to date. These entities are recognized as potential subjects for controlling the Cr(VI) movement in plants. Cr(VI) competes not only with sulfate transporters but also with sulfate assimilation pathway enzymes. This competition reduces the generation of methionine and cysteine, leading to the erroneous translation of essential proteins and ultimately causing S starvation. Prior research has documented that Brassica species, known as sulfur accumulators, tend to absorb much higher Cr. This implies that these plants utilize systems to absorb and transport Cr from the roots to the shoots. In addition, *Brassica rapa* and *Spinacia oleracea*, which tend to gather Fe, can also absorb elevated amounts of Cr and transport it to aboveground tissues [177]. Based on its sequestration in root cell vacuoles and subsequent bioaccumulation, most data have indicated a notable accumulation of Cr in plant roots. The plant xylem is the primary transporter of Cr after absorption [178]. Cr(VI) is carried throughout the endodermis of plants and then reduced to Cr(III), which is subsequently sequestered inside the cells of the root cortex. Examining Cr bioaccumulation in *Brassica juncea* under CrCl_3_ stress revealed that as the level of exposure to Cr rose, the volume of Cr residing in the cell wall, plastids, mitochondria, and nucleus increased significantly [179]. Cr sequestration predominantly occurs in plant roots since roots serve as the primary organ responsible for absorbing Cr from the soil. Moreover, Cr is considered the least mobile HM in plant roots [180]. The root level of Cr can be 100 times higher than in shoots. The presence of insoluble Cr forms is probably due to the increased Cr retention in plant roots. Nonetheless, several gene families have been identified as being in charge of moving metals from the roots to the shoots that are ATP binding cassette superfamily, HMA (heavy metal ATPase), NRAMP (natural resistance-associated macrophage protein), cation diffusion facilitator, and ZIP (ZRT, IRT-like protein) [181]. Nevertheless, despite their key functions in metal uptake, storage, transport, and resistance, our comprehension of transporter families to Cr treatment in plants remains incomplete. Further research is required to identify additional factors involved in the Cr transportation in model agricultural systems, in addition to sulfate transporters. This will enhance our comprehension of the spatial and mechanistic regulation of signaling pathways governed by Cr, hence expediting the advancement of Cr-tolerant crops in the future.

#### NPs alleviate cr stress in plants

##### Effects of NPs on cr absorption and movement in plants under Cr-induced toxicity

Plants do not yet possess a recognized transporter specifically designed for transporting Cr. Also, individual plants, Cr species, and the soil’s physicochemical properties impact the Cr behavior in the soil as well as its travel and accumulation in the organs. Metabolic inhibitors do not affect Cr(III) uptake yet reduce Cr(VI) uptake. This shows Cr(VI) absorption necessitates energy, while Cr(III) uptake does not require any. Thus, Cr(VI) absorption in roots is an active process involving the particular and non-specific channels responsible for transporting P, Fe, and sulfate ions [182]. This absorption is feasible because Cr(VI) bears a structural resemblance to these ions. Conversely, the absorption of Cr(III) might take place via osmosis, and the inhibitory effect of NPs on Cr uptake might be possible. Fe-NPs, for instance, have gained popularity as a reducing agent for environmental clean-up in recent years. Fe-NPs possess elevated surface energy and reactivity, making them suitable for the rapid decontamination of many substances, including highly toxic HMs [8]. The ability of Fe-NPs to remove HMs is mostly determined by the standard redox potential of HM contamination through processes of reduction and/or adsorption. Mohammadi et al. [183] showed that the use of 2% Fe-NPs had a substantial impact on reducing the translocation factor and bioaccumulation factor values of Cr in *Helianthus annuus*. By stopping sunflower plants from absorbing Cr, Fe-NPs help them grow better under Cr stress. The Fe-NPs greatly improve Cr immobilization, leading to a decrease in leachability, bioavailability, and bioaccumulation. Wheat plants at less than 50 mg/kg soil Cu-NPs decreased 60% and 58% in the amount of Cr in their shoots and roots, respectively, 7 days after planting. Furthermore, 30 days after sowing (DAS), the plants exhibited a significant drop of 59% and 52% in Cr levels in their shoots and roots, respectively. Cu-NPs can impede Cr mobility in soil, preventing plants’ absorption [184]. The Si-NPs led to a 40% reduction in the absorption of Cr in the root and a 36% decrease in transfer to aboveground organs [185]. The decrease was ascribed to the maintenance of the typical cellular arrangement in wheat plants under Cr stress. Nevertheless, CeO_2_-NPs caused a reduction in CrIII and CrVI species` accumulation in the roots and shoots [186]. The Cr accumulation reduction in sunflower plants might be due to CeO_2_-NPs` ability to limit Cr availability. Moreover, a substantial proportion of the CeO_2_-NPs is deposited in the cell membrane. Fe-NPs have shown the capacity to promote the development of sunflower plants in the presence of Cr stress by decreasing the uptake of Cr. The Fe-NPs were observed to greatly improve Cr immobilization, leading to a drop in its capacity to be washed out, its availability to living organisms, and its accumulation in plants. At 50 mg kg^− 1^ Cu-NPs in soil, 60% and 58% decreases were detected in Cr concentrations in the shoots and roots 7 days after sowing (DAS). Furthermore, 30 days after sowing (DAS), the plants exhibited a notable reduction of 59% and 52% in the Cr content in their shoots and roots, respectively. Cu-NPs can impede the mobility of Cr in soil, thereby preventing its absorption by plants [184]. Applying Si-NPs reduced Cr absorption in the root by 40% and its transit to aboveground organs by 36% [185]. This decrease was explained by the fact that Cr-stressed wheat plants still had their regular cellular structure. Ma et al. [186] investigated the effects of CeO_2_-NPs on sunflower plants subjected to Cr toxicity, and CeO_2_-NPs led to a reduction in the accumulation of both CrIII and CrVI species in both the roots and shoots of the plants. Moreover, a considerable amount of CeO_2_-NPs aggregated within the cell wall. They claimed that the ability of CeO_2_-NPs to reduce Cr availability is responsible for the decline in Cr accumulation in sunflower plants. This accumulation results in the binding of HMs, making them inaccessible and impeding their movement within the plant. Under 100 µM of Cr(VI), the shoots accumulated 62.5 mg/kg DW of Cr, and the roots accumulated 1472.6 mg/kg DW of Cr. However, when a combination of Si-NPs and Cr(VI) was treated, the shoots only accumulated 35.2 mg/kg DW of Cr, while the roots accumulated 516.6 mg/kg DW of Cr [187]. The rice seedlings accumulated 570 g/DW of Cr(VI) in the roots and 104 g/DW of Cr(VI) in the shoots under Cr(VI) stress. There was a significant decrease in accumulated Cr after the Si-NPs application. Specifically, Cr(VI) accumulation was 242 g/DW and 29 g/DW, respectively [188]. Fe-NPs decreased the absorption and accumulation of Cr in rice plants [189]. López-Luna et al. [190] found that applying up to 8000 mg/kg Fe_3_O_4_-NPs to wheat had no deleterious effect. It could enhance plant growth and decrease Cr and Cd in HM-stressed wheat plants. SiO_2_-NPs applied to *Brassica napus* seeds effectively decreased the Cr buildup in the roots and leaves [191]. They determined that SiO_2_-NPs enhance Si accumulation in the cells, which speeds up Cr deposition and Si binding in the cell wall. This process immobilizes Cr within the vacuoles, offsetting Cr accumulation in both the apoplast and symplastic areas. Similarly, SiO_2_-NPs applied to the leaves of *Brassica napus* at doses of 50, 100, and 150 µM effectively reduced the absorption in the roots and subsequent translocation to the leaves under Cr toxicity [192]. Kumar et al. [193] also investigated the impact of external TiO_2_-NPs on *Helianthus annuus* tolerance under Cr toxicity, and TiO_2_-NPs reduced Cr accumulation in both the roots and leaves. This reduction was followed by a corresponding decrease in TF (transfer factor) and BCF (bioconcentration factor) coefficients. The ZnO-NP applied for two types of chickpea plants also reduced Cr absorption in the roots and its movement when exposed to high levels of Cr. The decrease in Cr absorption might be due to the presence of ZnO-NPs, which operate as a physical barrier [194], along with elevated sorption capacity and expansive NP surface area. Furthermore, NPs show the ability to hinder roo shoot Cr transfer through alterations in both the physical composition and genetic activity.

##### Impact of NPs on the photosynthetic machinery in Cr-stressed plants

Cr stress reduces photosynthetic pigment levels and alters transpiration, water usage efficiency, and photosynthetic gas exchange rates in plants. In addition, Cr interacts with the hem groups of cytochrome by altering Cu and Fe`s redox state, leading to the inhibition of regular electron flow and the restriction of photosynthesis [188]. Furthermore, it demonstrates a significant ability to undergo oxidation and might overproduce ROS as an alternative electron-use route. This process ultimately hinders photosynthesis through an imbalanced redox mechanism [195]. The photochemical efficiency is only affected at high amounts of Cr dosage, and NPs have the potential to boost photosynthesis by optimizing the light-harvesting complex (LHC) and regulating the photosynthetic rate by targeted enzymes/genes, including carbonic anhydrase and RuBisCO [196].

Total chlorophyll, chlorophyll a, chlorophyll b, and carotenoid pigments were reduced by 62%, 46%, 30%, and 43%, respectively, when 200 mg/kg Cr was in the environment. However, 100 mg/L ZnO-NPs addition increased their contents by 14%, 4%, 7%, and 11%, respectively [197]. The detrimental effects of Cr(VI) on rice plants have similarly been demonstrated to be lessened by Si-NPs. The addition of Cr(VI) led to a 40% reduction in the total chlorophylls and a 29% drop in carotenoid concentration, as compared to the control; however, the addition of SiNPs led to an approximate 8% reduction in carotenoid concentration and a mere 10% reduction in total chlorophyll content. Cr(VI) exposure also led to a 28% and 34% decrease in Fv/Fm and qP, respectively. Nevertheless, the NPQ levels exhibited a 28% rise in comparison to the controls. Treatment with Cr + SiNPs resulted in a 5 and 3% decrease in Fv/Fm and qP, respectively, and a 9% rise in NPQ [188]. Si-NPs were added to pea seedlings, and photosynthetic metrics were improved when Cr(VI) was present. Adding a mixture of Si-NPs and Cr(VI) resulted in marginal reductions of 2%, 8%, and 3% in the values of Fv/F0, Fv/Fm, and qP, respectively. In contrast, plants treated solely with Cr experienced 15%, 24%, and 28% reductions in these values. In contrast, the NPQ demonstrated a 16% increase under Si-NPs + Cr(VI) treatment and a 37% increase during Cr stress over the controls [187]. Using ZnO-NPs also lessened the effects of Cr on the rice photosynthetic efficiency, provided possibly by antioxidants. Additionally, Fe-NPs have been suggested as a feasible way to lessen the accumulated amount of Cr and ameliorate stress. Fe-NPs (varying from 0 to 20 mg/kg) led to a rise in chlorophyll content, despite the simultaneous surge in Cr level from 0 to 0.1 g/kg. Fe-NPs led to improvements in the photosynthetic rate, gs, and WUE in plants treated with Cr [189]. Singh et al. [194] discovered that incorporating ZnO-NPs successfully alleviated the detrimental impacts of Cr by improving the photosynthetic characteristics in chickpeas subjected to Cr-induced stress. ZnO-NP promoted pigment stability and enhanced overall photosynthesis by mitigating oxidative damage and lowering Cr uptake. *Helianthus annuus* plants were damaged by Cr stress, which closed the stomata and reduced the gas exchange parameters. Concurrent with this, the chlorophyll a and b pigments, the Fv/Fm values, and the carotenoid content all decreased. TiO_2_-NPs under Cr stress did, however, protect stomatal and epidermal guard cells, yielding advances in gas exchange parameters, photosynthetic pigments, and Fv/Fm rates and according to Huang et al. [192], using Si-NPs improved gas exchange and pigments that help plants when stressed by Cr. Furthermore, Si-NPs aided in preserving photosystem PSII’s functionality and mesophyll cells’ ultrastructure. The improvement in photosynthesis resulting from Si-NPs can be attributable to the amplified efficacy of carbon absorption or the facilitation of light reactions, both of which trigger photosynthetic stimulation. Ulhassan et al. [191] demonstrated that treating *Brassica napus* seeds with SiO_2_-NPs optimized the genesis of photosynthetic pigments by activating the expression of *CAO*, *POR*, and *CHLG*, which are involved in chlorophyll synthesis. Additionally, this treatment delayed the leaf senescence by suppressing the expression of *SAG12* during Cr stress. Ma et al. [186] and Noman et al. [184] demonstrated that CeO_2_- and Cu-NPs modify pigment levels and improve photosynthetic machinery functioning in sunflower and wheat, respectively.

These results indicate that Cr stress leads to a considerable decrease in photosynthetic parameters. And NPs greatly improve these factors. This might be because they stop Cr from being absorbed and moving around the cells, which turns on antioxidant enzymes and lowers oxidative stress. Therefore, NPs can impact the pace of photosynthesis by regulating specific genes and enzymes that play critical roles in photosynthesis [163]. However, to date, no study has been conducted to explicitly analyze the influence of NPs on these enzymes in the presence of Cr stress.

##### The role of NPs in strengthening the antioxidant defense system during cr toxicity

NPs can keep the balance of ROS or boost the activity of antioxidants in stressed plants. Applying Cu-NPs to wheat plants increased cellular antioxidants by making CAT and POD more active, and also proline concentration and total phenolic content were increased in Cr-stressed plants. The observed impact was absent in plants subjected to Cr stress, however, not treated with Cu-NPs. Noman et al. [184] suggested that higher levels of antioxidant defense system components may be linked to reduced Cr translocation (from the soil to plant upper portions). The SOD, POD, APX, and CAT in rice plants were also reported to be significantly enhanced by increments in the concentrations of Fe-NPs from 0 to 20 mg/kg [189]. Additionally, adding 100 mg/L ZnO-NPs decreased the antioxidant enzyme activity in wheat plants at a dosage of less than 200 mg/kg Cr [197]. On the other hand, applying 25 µM ZnO-NPs to rice plants treated with Cr raised the activity of antioxidant enzymes (GR, APX, DHAR, and MDHAR), which are linked to the AsA-GSH cycle [198]. ZnO-NPs, at 25 µM and 20 nm in size, increased the activity of antioxidant enzymes Singh et al. [194]. This made the antioxidant capacity of bean plants better under Cr toxicity. TiO_2_-NPs have also been shown to have the ability to decrease ROS levels in Cr-stressed *Helianthus annuus* plants via enhancing APX, SOD, MDHAR, DHAR, and GR performance. In addition, using Si-NPs increased the activity and expression of antioxidant enzymes (CAT, SOD, POD, and APX). This lowered the H_2_O_2_ and superoxide anions in *Brassica napus* under Cr stress and helped the plants adapt by protecting bio-macromolecules like membrane lipids [192]. Ulhassan et al. [191] also found that Si-NPs nano-primed *Brassica napus* seeds improved the antioxidant defense system when Cr was present. Furthermore, sunflower plants’ defense system and ability to respond to Cr toxicity were improved by CeO_2_-NPs (25 and 50 mg/L), which proved to be effective by enhancing antioxidant enzymes and EL [186]. Tripathi et al. [187] discovered that the increased levels of APX and SOD in reaction to Cr(VI) stress were not enough to adequately protect pea seedlings from overgenerated ROS, as evidenced by the higher levels of MDA. However, when Cr(VI) was paired with Si-NPs, it either improved the way APX and SOD function better or made Cr(VI) less of an inhibitor for GR, CAT, and DHAR activities.

We may deduce that NPs may mitigate plant Cr toxicity and accumulation via several pathways (e.g., increasing the soil’s antioxidant capacity, promoting photosynthesis, increasing mineral intake for plant nutrition, and immobilizing Cr in the soil or functioning as nano-fertilizers) by metal and metal oxide NPs in plants subjected to Cr stress.

##### Effects of NPs on the accumulation of micro- and macro-nutrients under cr toxicity

Insufficient quantities of macro and micronutrients may impede the development of plants and diminish yield since Cr inhibits the absorption of both macro and micronutrients. This is because Cr structurally resembles other crucial ions and hinders root penetration and growth [196]. The shoots of plants treated with Cr revealed a 17% reduction in Si content. On the other hand, Sharma et al. [188] discovered that using Cr + Si-NPs increased the Si content in shoots by 33%. The total amount of Zn in wheat grains, roots, and shoots lowered to 87%, 75%, and 71%, respectively, when treated with 200 mg/kg Cr. Adding 100 mg/L ZnO-NPs to a treatment of 200 mg/kg Cr raised the amount of Zn in the grains, shoots, and roots by 80%, 58%, and 61%, respectively [197]. Thus, NPs help Cr-stressed plants take in more macro and micronutrients. Additionally, the 100 µM Cr(VI) greatly lowered the amounts of Ca (56% and 11%), Mg (15 and 17%), P (6% and 34%), and K (9% and 33%) in the shoot and roots of hydroponically grown *Pisum sativum*. Compared to treatment with Cr(VI) alone, the Si-NPs + Cr(VI) made Cr(VI) ‘s adverse impacts on micro- and macronutrient accumulation much less evident [187]. Ulhassan et al. [196] showed that SiO-NPs before Cr stress in Rapeseed seeds increased the absorption of Zn (40/44%), Fe (41/43%), Ca (35/40%), K (37/35%), P (45/39%), and Mg (47/38%) in the leaves and roots. Adding SiO_2_-NPs before the mineral nutrients most likely improves their effectiveness by lowering the damage that Cr causes to photosynthesis, the cellular membrane (lipid peroxidation), and the antioxidant defense system. Consequently, this stimulation enables the Rapeseed tissues to gather more mineral nutrients. However, the exact mechanism responsible for this event is still unexplained. There may be a link between the NPs combined with Cr stress and the increased root development resulting from reduced absorption and Cr transfer from the soil to plant parts.

### Impacts of nanoparticles on plants under other heavy metal toxicity

Excessive deposits of Cu, Al, Ni, and Zn are a major environmental problem with negative consequences for all life forms. Cu, for instance, is a typical transition metal in the Earth’s crust valued for its heat and electrical conductivity qualities. Despite being a vital microelement for plants and a cofactor in biochemical and physiological processes, at higher-than-optimal levels, Cu quantities cause cellular toxicity by preventing the uptake of other elements, oxidative stress, cell damage, and lowering levels of pigments [199, 200, 249].

Plants show obvious toxicity signs when subjected to higher Cu, such as slow growth or even death [199]. Several studies have indicated that using NPs can be a viable strategy for reducing plant Cu toxicity. Growing *Brassica napus* on an MS containing 5 mg/L Cu resulted in a decrease in growth and biomass, a rise in Cu accumulation, and oxidative stress development [201]. Less Cu accumulation and more K, Mn, Mg, and Ca after 300 mg/L of S-NPs were added to the Cu-containing MS was detected, and this treatment also increased POD, SOD, GST, GR, and CAT enzymatic activity in *Brassica napus.* Using carbon NPs, on the other hand, improved germination and germination index and decreased germination time in Cu-stressed maize. Xin et al. [202] found that positive effects occurred in conjunction with elevated root and stem CAT, POD, and SOD activities and decreased Cu content. Exposure to non-toxic amounts of TiO_2_-NPs (10 mg/L) at the same time as Cu concentrations of 1 and 2 mg/L increased the toxicity and deposition of both Cu and Ti in soybean seedlings, stopping Cu from moving from roots to shoots [203]. The interaction effects became minimal when the Cu level for simultaneous exposure surged or equalled 5 mg/L. After 48 h of simultaneous exposure, the absorption of Cu ions on TiO_2_-NPs rose progressively from 31 mg/L to 118 mg/L, corresponding to a rise in Cu level from 1 to 20 mg/L. TiO_2_-NP deposition increased significantly after 48 h of simultaneous exposure to Cu concentrations of more than 5 mg/L, compared to TiO_2_-NP exposure alone. Higher TiO_2_-NPs deposition in soybeans might reduce Cu’s bioavailability, thereby relieving Cu toxicity by TiO_2_-NPs. The data show how important it is to understand TiO_2_-NPs` effect on the phytotoxicity of HMs, especially Cu and TiO_2_-NPs and metals interaction, ending in risk estimates. Konate et al. [204] stated that exposure to Cu stress caused a decline in the development and biomass of wheat seedlings. Specifically, 10 mM resulted in seedling death. Adding 200 mg/L of Fe-NPs helped wheat plants adapt to Cu toxicity. The beneficial impacts of Fe-NPs may be ascribed to the heightened functionality of POD and SOD, in addition to the enhancement of plant antioxidant capacity. Xin et al. [205] found that the influence of polysuccinimide (PSI)-NPs on maize seed germination was dosage dependent under different degrees of Cu stress, with the best impact observed at 200 mg/L. Better shoot and root development and higher enzyme activities were signs that PSI-NPs were present and had successfully reduced Cu toxicity. The moderating impact of PSI-NPs can be credited to the higher functionality of antioxidant enzymes and the containment of Cu as Cu-PSI complexes, leading to a decrease in phytotoxicity.

Crop production in acidic soils around the world is greatly limited by toxic Al. Soil pH falls below 5 triggering the production of Al^3+^, which enters root tip cells and stops root growth. Al becomes the principal element that causes phytotoxicity in acidic soils with a high mineral concentration [206, 250]. Al toxicity can harm the development and general performance of several plant species [158]. NPs are one of the most effective methods currently available to reduce Al toxicity in plants. Using TiO_2_-NPs, Mariz-Ponte et al. [158] showed that lettuce grown under Al stress benefited in several ways, such as improved germination, water content, seedling length, membrane potential balance, stomatal conductance, intercellular CO_2_, and net CO_2_ assimilation rates. The efficiency of photosystem II was also enhanced, and the anthocyanin levels were elevated in lettuce plants treated with TiO_2_-NPs. In addition, Al promoted starch accumulation at the expense of soluble sugar synthesis. Nevertheless, these effects were partially reduced by adding TiO_2_-NPs. Two *Glycine max* cultivars were subjected to ZnO-NPs treatments (25 to 150 mg/kg) and Al (0.3 g/kg) and, using ZnO-NPs, significantly improved growth and photosynthetic pigments` intactness. The increase in performance can be explained by the improved SOD function, corresponding to less damage to the plasma membranes reflected by lower MDA levels [207]. The effect of chemically and biogenic Si-NPs on *Cicer arietinum* plants to Al stress showed that by reducing the accumulation of H_2_O_2_ and superoxide anions [208], both types of Si-NPs modified the activity and expression of CAT, APX, and SOD enzyme genes. As a result, plants were able to better respond to Al stress, and reducing Al stress was a successful outcome for both chemically and biogenically produced Si-NPs. In contrast to the chemically produced NPs, the green Si-NPs were shown to alleviate damage symptoms more effectively, even at lower concentrations. Si-NPs in combination with Al lowered the activities of NADPH oxidase and photorespiratory enzymes in Al-exposed maize plants [209] and enzymatic and non-enzymatic (AsA, GSH, flavonoids, polyphenols, and FRAP) antioxidant defense mechanisms were both strengthened. Metal detoxification (by GST in particular) and organic acid accumulation in the roots were both improved by Si-NPs. The protective mechanisms induced by Si-NPs showed effects unique to certain organs and dependent on the amount of Al administered. The results collectively provide comprehension of the cellular and metabolic pathways. Yet, NPs may be utilized to mitigate Al stress in acidic soils, suggesting a new approach that still requires more research.

While small amounts of Zn and Ni are essential for plants to grow, at higher concentrations they become toxic. High levels of Ni and Zn can cause phytotoxicity by inhibiting growth and reducing relative water content. These two also affect various aspects of photosynthesis, pigmentation, osmolality, and enzyme activity, resulting in leaf chlorosis and necrosis [210, 211]. In a 2-year field experiment, El-Saadony et al. [212] investigated the efficacy of Si-NPs (2.5 and 5.0 mmol/L) spray on *Phaseolus* vulgaris grown in Ni-polluted soil. Their research showed that Si-NPs application resulted in significant beneficial effects on several aspects of growth and plant performance, which included improvements in chlorophyll and carotenoid levels, net photosynthesis, transpiration, stomatal conductance, membrane stability index, free proline levels, total soluble sugar levels, and increased P, N, K, Ca and enhanced POD, CAT, APX, and SOD activities. Using Bio-Si-NPs significantly reduced EL, MDA, H_2_O_2_, O_2_^−^ and Ni levels in *Phaseolus vulgaris*. Konate et al. [204] showed that exposure to Zn stress induced a significant reduction in root length, shoot length, and activity of SOD and POD while increasing MDA levels. Fe-NPs (2000 mg/L) effectively reversed growth inhibition and initiated protective responses to counteract oxidative damage caused by Zn stress. Another study assessed the role of Fe-NPs on Zn accumulation in castor plants under Zn stress conditions. Fe-NPs could affect the production of starch granules during Zn stress, and SEM revealed distinct structural changes in the phloem and xylem when Fe-NPs were introduced. At the soil aggregate level, changes in the size distribution of the soil structure were observed following the introduction of Fe-NP, and the macro aggregates and the clay fraction increased, whereas micro aggregates decreased. Therefore, Fe-NPs might alter the distribution and movement of Fe and Zn between the different soil aggregate size fractions, clay, and macroaggregates. Furthermore, the migration of Fe and Zn was reciprocal, and the content of Zn in castor organs, including roots and shoots, was influenced by Zn concentration in the larger aggregate portions [213].

## NPs-based toxic effects on heavy metal stress in plants and influencing factors

Nanoparticles can cause changes in both biological and non-biological systems when exposed to HMs. The changes are determined mainly by the concentration and physical attributes of the NPs, with the shape, size, and surface charge being the most significant aspects. The characteristics of soil, such as pH, moisture content, organic matter content, cation exchange capacity, and texture, have the power to affect the reactivity, fate, and hence NP toxicity [253]. In HM-polluted soils, metal NPs can experience a range of biological, chemical, and physical processes that impact the metal availability and, subsequently, the level of toxicity. NPs in natural settings undergo many processes, including excretion, absorption, retention, accumulation, precipitation, dissolution, transformation, assimilation (digestion-ingestion) by organisms, and interactions with other molecules [254]. Most of these reactions are sensitive to the pH level. Alkaline conditions facilitate the aggregation of metal NPs, while acidic conditions cause metal NPs to convert into ionic forms quickly. Barley plants have higher toxicity to CuO-NPs when exposed to acidic environments, accompanied by an elevated Cu release from the NPs [255]. ZnO-NPs elicit contrasting responses in alkaline (pH 8) and acidic (pH 5) soils. In alkaline soil, ZnO-NPs promote plant germination and development; however, in acidic environments, they have detrimental effects [256]. The diverse clustering resulting from the pH-dependent interactions between metal NPs and soil components enhances their spatial and electrostatic stability but hinders their movement and distribution in the soil. Aggregation reduces the amount of particle surface area that is exposed, resulting in lower rates of dissolution and ion release, which can help minimize the harmful effects of such particles on biological systems [257].

Apart from the intrinsic properties of NPs and the physico-chemical characteristics of the soil, other factors impact the effects of NPs on HM-stressed plants. For instance, low and high-molecular-weight organic molecules in root exudates (amino acids, fatty acids, polysaccharides, metal ions, etc.) can alter the rhizosphere environment, microbiome, and ultimately the fate of metal-NPs [251]. Indeed, NPs release metal ions, adhere to or deposit onto root surfaces, and even undergo chemical alterations due to acids and oxidizing/reducing substances of exudates [22, 258]. Cu-NPs and Fe-NPs form hydroxide precipitates, which are not accessible to plants due to exposure to root exudates. Moreover, the decrease in soil pH resulting from acidic root exudates of soybean plants leads to the conversion of ZnO-NPs into Zn ions and Zn-citrate in the rhizosphere [258]. Additionally, metallic NPs on the root surface can alter root exudates, root surface chemistry, and rhizosphere`s microbial community, which impact root nutrient and HM uptake and soil properties. It has been demonstrated that PGPRs in the rhizosphere of *Salvia miltiorrhiza* were induced by the application of CuO-NPs [259], whereas Ag-NPs application induced changes in root exudates of mustard, chickpea, and wheat, leading to the abundance of rhizosphere diazotrophic bacteria [260, 261]. Se-NPs enhanced the populations of beneficial microorganisms belonging to *Anaerolineae*, *Deltaproteobacteria*, *Gemmatimonadetes*, *Bacteroidia*, *Alphaproteobacteria*, and *Gammaproteobacteria* in the rhizosphere. This study revealed a significant correlation between alterations in the microbial community and factors such as soil metabolites, enzymes, environment index, and Se forms. These factors were found to decrease the Cd bioavailability and accumulation in pepper plants [262]. Fe_3_O_4_-NPs and TiO_2_-NPs increase the levels of methionine and cysteine and cause changes in P composition in the exudates of wheat and lettuce roots [263, 264].

In addition to the beneficial effects and properties of NPs, certain characteristics such as long-term persistence, low biodegradability, and continuous deposition can negatively impact plants and soil organisms. These effects can be exacerbated in environments contaminated with HMs, potentially intensifying plant toxicity. Therefore, the phytotoxicity assessment of NPs is a crucial prerequisite for promoting nanotechnology applications and mitigating potential ecological risks, particularly in soils contaminated by toxic metals. Typically, NPs harm plant performance when used at concentrations that exceed what is favorable either to growth or response to natural circumstances or HM stress unless there are certain exceptions [265]. This group of NPs emphasizes certain types, e.g., Cu-NPs and Zn-NPs. This is because the quantities of these elements that might cause toxicity or favorable fertilizing effects are pretty close, and this is determined mainly by the type of plant and soil qualities [266, 267].

The NPs at high concentrations cause damage to various cellular organelles, leading to excessive ROS and oxidative stress induction at the cellular level. This disruption affects the integrity of the cell membrane, damages enzyme activity, reduces photosynthesis processes, and triggers genotoxic effects [253]. Soil pH is one of the crucial factors influencing the toxicity of metal-NPs. The impact of acidic and alkaline pH on the growth of two tomato and bean plants, which were exposed to a range of ZnO-NPs (ranging from 3 to 225 mg/kg), was examined, and the findings revealed that plants experienced total degradation in acidic soil when exposed to higher concentrations of ZnO-NPs. On the other hand, tomato and bean plants sustained their usual growth rates in alkaline soils, even when exposed to multiple levels of ZnO-NPs [268]. Excessive NP buildup in the soil may disrupt or modify the microorganism population in the soil microbiota, which in turn affects the breakdown of organic compounds and nutrient recycling [252]. Studies have shown that TiO_2_-NPs delay the nodule formation and specific changes in the pea root structure. This ultimately results in a decrease in the formation of a mutually beneficial relationship between legumes and rhizobium bacteria, as well as damage to the outer surface of *Rhizobium leguminosarum cells* [269]. Moreover, scientific evidence has shown that ZnO-NPs, in low concentrations ranging from 25 to 400 mg/kg, do not cause any detrimental impacts on the mutually beneficial relationship between mycorrhizal fungi and tomato or maize plants [270, 271]. Nevertheless, larger amounts (from 500 to 3200 mg/kg) induce a decline in the mycorrhizal symbiosis in maize [271, 272].

The utilization of NPs in environments contaminated with HMs under certain conditions may lead to unforeseen increases in toxicity or the accumulation of toxic metals or NPs within plants, which could exhibit more potent (synergistic), comparable (additive), or even weaker (antagonistic) effects compared to what would be predicted based solely on the toxicity of HMs. The primary factors affecting the enhanced induction by NPs in HM toxicity include plant species, inherent properties of NPs and toxic metals, experimental and environmental conditions (soil natural or hydroponic environments), and the method of NP treatment application (foliar spray, direct soil application, seed priming, etc.). The mechanisms underlying toxicity induction resulting from the co-exposure of NPs and HMs are highly intricate. Given the limited data in this area, a comprehensive and precise understanding is currently lacking, especially in natural soil conditions. Nevertheless, various processes can occur concurrently or independently, encompassing alterations in their breakdown or availability, changes in absorption, translocation, and internalization within plants, as well as modifications in metabolic processes involved in detoxification and excretion processes from the rhizosphere or within the plant [273, 274]. Zhang and Zhang [213] stated that the exacerbation of toxicity resulting from co-exposure to NPs-HMs may stem from the altered availability of toxic metals in plants. Considering NPs characteristics (high absorption capacity, large specific surface area, and high reactivity), if HMs are trapped within precipitating NP aggregates or are absorbed by NPs, the bioavailability along with their bio-accumulation will decrease [275]. Indeed, HMs can potentially induce changes in the functional groups of NP coatings or their surface properties, thereby leading to the generation of heterogeneous or homogeneous NP aggregates. These processes ultimately impact HM toxicity by reducing metal-NP dissolution and the release of metals [276]. Metallic NPs and the metals they release in the rhizosphere might compete with HMs for active absorption sites, impacting their bioavailability [273]. Furthermore, studies have shown that NPs might indirectly affect the dispersion and, eventually, the accessibility of HMs by modifying soil aggregate formation [213].

In the rhizosphere, HMs are absorbed onto the surface of NPs, which can lead to dual outcomes. Indeed, NPs can act as carriers for HMs, facilitating their entry into cells, a phenomenon known as the Trojan horse type entry. Therefore, HMs transfer into the plant and subsequent metal release can potentially exacerbate the toxicity [273]. Also, under certain conditions, NPs potentially enhance the conversion of toxic compounds into more toxic forms or reduce the rate of internal degradation of these compounds, thereby increasing the toxicity [274].

Most studies for NPs under toxic HM have focused on the positive role of this interaction [277], primarily stemming from factors such as short exposure durations, hydroponic culture media usage, selection of appropriate concentrations through pilot experiments, and so forth. Nevertheless, experiments have reported that NPs can exacerbate HM toxicity in various plants (Table 5). Banerjee et al. [278] demonstrated that biogenic ZnO-NPs, up to a concentration of 300 mg/L, in two separate experiments, positively impacted germination and seedling growth of *Pisum sativum* under As toxicity. However, surpassing ZnO-NPs concentration up to 400 mg/L was associated with reduced germination accompanied by the induction of cytotoxicity and toxic molecular effects on DNA and chromosomes. According to another study, when the concentration of ZnO-NPs was raised to 200 mg/L in seed priming, there was an increase in H_2_O_2_ and superoxide anion formation and a drop in relative water content. As a result, oxidative stress levels went up in blackgram plants subjected to As. Since the lowest accumulation of As in both roots and leaves was observed in plants treated with ZnO-NPs, the exacerbation of As toxicity under the treatment of 200 mg/L ZnO-NPs was attributed to an excessive accumulation of Zn ions in the plants compared to other treatments (As treatment alone or As treatment combined with different ZnO-NPs levels) [279]. Haisel et al. [280] demonstrated that even at low concentrations (10–50 µM), ZnO-NPs exacerbated Cd toxicity in *Carex vulpina*, accompanied by increased Cd accumulation and reduced photosynthetic pigments. Applying TiO2-NPs at low Cu concentrations (1 to 2 mg/L) in soybean plants dramatized Cu toxicity. However, when the Cu content in the Hoagland medium increased (5–20 mg/L), the administration of TiO_2_-NPs boosted plant defense against Cu toxicity. Indeed, when exposed to high concentrations of Cu, a substantial amount of Cu became attached to the surfaces of TiO_2_-NPs. This enabled the Cu to settle and stick together, decreasing the availability of this metal to the plant. Consequently, there was a drop in the accumulation of both Cu and Ti in the plant [203]. Similarly, under hydroponic conditions, Ni lower than 1 ppm led to an increase in Ni accumulation and a reduction in the biomass of *Sorghum bicolor* with ZnO-NPs. However, higher Ni concentrations up to 5 ppm, resulted in decreased Ni accumulation and improved plant growth by ZnO-NPs [281]. Therefore, NP application methods can be a crucial determinant in shaping plant responses to NPs treatment under HM toxicity. Lian et al. [282] examined the impact of TiO_2_-NPs at concentrations of 100 and 250 mg/L, applied via root surface or foliar exposure, for Cd toxicity in maize plants. The findings revealed that root exposure worsened the toxic effects of Cd, yet foliar exposure only partially amplified Cd toxicity at higher concentrations of TiO_2_-NPs. However, the concentration of 100 mg/L improved the plant’s ability to tolerate Cd toxicity. The simultaneous application of Ag-NPs and antimony (Sb) led to a higher accumulation of Sb and Ag in soybeans, which was associated with increased ROS accumulation, thereby inducing oxidative stress. However, plants’ accumulative response of the two forms of Sb (III/V) to Ag-NPs was different [283]. The plant’s growth stage has also been recognized as one of the factors influencing the effects of NPs under HMs stress. For example, Gonzalez-Moscoso et al. [284] demonstrated that the application of Si-NPs led to a decrease in As-exposed tomato yield, while at the seedling stage, it had a positive effect on plant growth. Zhang et al. [285] demonstrated that ZnO-NPs enhanced the ability of rice to cope with Cd toxicity in the seedling and tillering phase; however, they did not observe any beneficial effects of ZnO-NPs on rice yield and growth during the heading stage under Cd stress.

**Table 5:** The nanoparticle (NP) toxicity effects in plants under heavy metal (HM) stress

HM	NPs			Plant species	NP-toxicity effects	Ref.
	Type	NP-toxicity concentration	Application method			
As	ZnO-NPs	400 mg/L	Aqueous suspension, soil	*Pisum sativum*	Reduction in germination and plant growth, induction of oxidative stress, decrease in enzyme activity, induction of genotoxic effects, and instability in chromosomes	[278]
		200 mg/L	Seed priming	*Vigna mungo*	Increase in H_2_O_2_, superoxide anion, and MDA accumulation, reduction in relative water content, and induction of oxidative stress.	[279]
		100 mg/L	Hydroponic cultivation	*Oryza sativa*	Decrease in biomass and total chlorophyll, increase in electrolyte leakage	[14]
	SiO_2_-NPs	250 and 1000 mg/L	Soil	*Solanum lycopersicum*	Decreased plant yield	[284]
Cd	ZnO-NPs	10 and 50 µM	Hydroponic cultivation	*Carex vulpina*	Increase in Cd and Zn accumulation in roots and leaves, reduction in photosynthetic pigments.	[280]
		500 mg/kg	Soil	*Phytolacca americana*	Damage to root cells, reduction in root and shoot growth, induction of oxidative stress.	[276]
		250 and 500 mg/kg	Soil	*Sorghum bicolor*	Inhibition of soil enzyme activity, reduction in root and shoot biomass, increase in Zn accumulation in the plant	[286]
		500 mg/kg	Soil	*Oryza sativa*	Increased Cd in root, shoot, and grain	[285]
	TiO_2_-NPs	100 and 250 mg/L	Foliar exposure, hydroponic cultivation	*Zea mays*	Increasing accumulation of Ti and Cd in roots and shoots and induction of oxidative stress	[282]
	CuO-NPs	20 mg/L	Hydroponic cultivation	*Brassica*	Increase in Cd accumulation, induction of oxidative stress, reduction in nutritional value	[287]
Cu	TiO_2_-NPs	10 mg/L	Hydroponic cultivation	*Glycine max*	Increased Cu toxicity at 1–2 mg/L Cu, yet improved plant tolerance at higher Cu concentrations (5–20 mg/L)	[203]
Ni	ZnO-NPs	50 and 100 ppm	Hydroponic cultivation	*Sorghum bicolor*	Increased Ni accumulation in low Ni concentration, increased Ni accumulation, and improved plant growth under high Ni concentration	[281]
Sb (antimony)	Ag-NPs	1 mg/L	Hydroponic cultivation	*Glycine max*	Increase in Sb accumulation and production of ROS and induction of oxidative stress, decrease in photosynthetic pigments	[283]

Although the positive impacts of NPs have promoted their use in enhancing agricultural yield and sustainable farming, it is crucial to consider the negative consequences and toxicity that may arise from their application. Based on the discussions in this section, the impact of applying NPs to counteract the toxicity of HMs can be either positive or negative. The outcome is influenced by several factors, such as the specific type and concentration of NPs and HMs, the composition of the growth medium, the method of treatment application, the duration of exposure, the stage of plant growth, and the particular plant species involved. The primary risk factor to consider is the possibility of synergistic interactions between NPs and HMs. A prominent constraint in employing NPs in agriculture is the challenge of comparing findings among research done under diverse experimental settings, which might deviate substantially from complex natural scenarios. Therefore, it is essential to use a systematic strategy with well-defined procedures that explicitly outline as many pertinent elements as possible. Furthermore, there is a lack of knowledge regarding the actual collective impacts of mixtures, including both NPs and HMs. This emphasizes the need for further investigation to comprehend better how NPs and HMs interact. This should include contrasting analyses with their ionic forms and bigger sizes. In the near future, the use of NPs will help enable the adoption of sustainable agriculture by reducing the reliance on external inputs and limiting the presence of chemical residues in crops.

## Conclusions and future perspectives

HM stress causes diverse impacts on plants with the potential to significantly decrease necessary metal absorption and the triggered oxidative burst by HM accumulation [288, 289]. This process is contingent upon several critical variables, including the nature of NPs and HMs, the soil’s physio-chemical features, the plant species involved, and the approach used for NP application. Various techniques for metal scavenging focused on NP have been documented in the context of plant-soil interaction. These methods focus on optimizing the soil’s ability to immobilize toxic metals, improving how plants absorb and distribute these metals, and triggering plants’ natural defense mechanisms against oxidative stress by upregulating the expression of genes that enhance a plant’s tolerance. Considering that NP-based technology for optimizing HM presence in plants and soils is still in its infancy, more research is needed to examine NP-oriented methods for plant HM absorption and HM immobilization in soils.

Research on mitigating HM stress has shown that the concentration of NPs and the various ways of administration have varying effects on plants. The issue at hand is the lack of considerable control impact at low dosages, while high dosages might lead to negative consequences on plants or wasteful expenses. Furthermore, there are still uncertainties surrounding the application strategies of NPs in the soil environment. Therefore, more study is necessary into the procedures and curative effects of different application strategies [290, 291]. Also, using different NPs in combination with other chemicals is an innovative field of research. Additional investigation into the potential synergistic effects of NPs with biochar, NO, H_2_S, phytohormones, and HM-resistant microbial strains is required. Understanding the molecular underpinnings of the collaborative interactions between plants, NPs, and HMs is crucial for deciphering the processes involved in managing HMs, promoting plant development, and abiotic tolerance. The mechanism by which NPs affect cellular antioxidants, resulting in improved plant tolerance to stress, remains uncertain. Smarter designer NPs that reduce stress and boost sustainable agricultural output can be developed with a better understanding of these processes.

Although nanotechnology offers promising solutions for dealing with the toxicity of HMs, the fate of the interaction between HMs and NPs in the natural environment is uncertain, while investigating the effects of this interaction on ecosystems is essential. Excessive use of some antifungal and antibacterial NPs can disrupt the ecological equilibrium of water and soil [292, 293]. Processes such as aggregation are vital in determining the fate of NPs and affect their reactivity, toxicity, and mobility. Aggregation increases particle size and reduces surface area, making NPs less mobile and reactive, thereby reducing potential undesirable effects in nature. Future research also needs to focus on discovering the hazards and behavior of residual NPs in the environment and their interaction with HMs. Furthermore, creating environmentally compatible NPs that can decay naturally is crucial for ensuring sustainable and safe remediation solutions.

The cost of NPs is a significant barrier hindering their widespread adoption in agriculture. Hence, the efficient, environmentally friendly, and cost-effective synthesis of NPs is crucial to advancing nanotechnology. Furthermore, the integration of big data paired with artificial intelligence technology is already underway in the field of environmental research, as well as in other fields. Through the accumulation of experimental data and advancements in technology, it is now possible to forecast the physiological characteristics of plants and HMs in plants by analyzing the properties of soil, NPs, and plant species. One can choose suitable types and concentrations of NPs, minimizing superfluous expenses and optimizing the positive impact.

## Data Availability

No datasets were generated or analysed during the current study.
